# Whole genome sequencing of canids reveals genomic regions under selection and variants influencing morphology

**DOI:** 10.1038/s41467-019-09373-w

**Published:** 2019-04-02

**Authors:** Jocelyn Plassais, Jaemin Kim, Brian W. Davis, Danielle M. Karyadi, Andrew N. Hogan, Alex C. Harris, Brennan Decker, Heidi G. Parker, Elaine A. Ostrander

**Affiliations:** 1 Cancer Genetics and Comparative Genomics Branch, National Human Genome Research Institute, National Institutes of Health, Bethesda, MD 20892 USA; 2 Present Address: Texas A&M University, College Station, TX 77840 USA; 3 Present Address: Laboratory of Genetic Susceptibility, National Cancer Institute, National Institutes of Health, Rockville, MD 20850 USA; 4 Present Address: Department of Pathology, Brigham and Women’s Hospital, Harvard Medical School, Boston, MA 02115 USA

## Abstract

Domestic dog breeds are characterized by an unrivaled diversity of morphologic traits and breed-associated behaviors resulting from human selective pressures. To identify the genetic underpinnings of such traits, we analyze 722 canine whole genome sequences (WGS), documenting over 91 million single nucleotide and small indels, creating a large catalog of genomic variation for a companion animal species. We undertake both selective sweep analyses and genome wide association studies (GWAS) inclusive of over 144 modern breeds, 54 wild canids and a hundred village dogs. Our results identify variants of strong impact associated with 16 phenotypes, including body weight variation which, when combined with existing data, explain greater than 90% of body size variation in dogs. We thus demonstrate that GWAS and selection scans performed with WGS are powerful complementary methods for expanding the utility of companion animal systems for the study of mammalian growth and biology.

## Abstract (web summary)

Being man’s best friend, dogs have been bred and selected for certain morphologic traits and breed-associated behaviours. Here, Plassais et al. analyse 722 canine whole genome sequences including modern breeds, wild canids and village dogs by GWAS and search for signatures of selection.

## Introduction

Most domestic dog breeds (*Canis lupus familiaris*) were developed within the last two-hundred years as a result of direct selection designed to fulfill working or aesthetic requirements^1–4^. Today, 193 breeds are registered by the American Kennel Club (akc.org/dog-breeds/)^5^, and 360 recognized internationally by the Fédération Cynologique Internationale (fci.be/nomenclature/). Breed creation is typically initiated by reproductively isolating a small number of homogeneous founder animals with specific characteristics, or alternatively, founders from multiple breeds with desired phenotypes are combined^4,6,7^. In either paradigm, population bottlenecks and popular sire effects frequently reduce breed genetic diversity, with potentially deleterious effects^8,9^. Thus, stringent selections for morphological and behavioral characteristics have produced an inimitable system for identifying genetic variants and understanding their biological consequences on mammalian traits and disease susceptibilities.

The same selective pressures that reduced phenotypic and genotypic heterogeneity within breeds^8,10,11^ result in long stretches of intra-breed linkage disequilibrium (LD)^1,7,12^. Inter-breed LD is shorter and further reduced as breed relatedness decreases^4^. This unique genomic-demographic architecture has facilitated the study of dog breeds, leading to the identification of genes underlying both simple and complex morphologic traits^13–18^. Additionally, the dog model has been utilized to identify genes with translational potential for human health and biology, including both rare and common human disorders, such as autoimmune disease, neuromuscular disorders and cancer^19–22^. To date, most canine genome-wide association studies (GWAS) utilized one or small numbers of breeds analyzed with the Illumina Canine HD SNP array which contains 172,115 SNPs. At this variant density, associated haplotypes at any locus may extend for kilobases to megabases (Mb). At the extreme, this can impede the identification of causal variants. While utilizing multiple breeds that likely share a common ancestry may facilitate the reduction of haplotype length, they often lack the granularity to implicate a single gene for follow-up, much less a single variant.

We develop a data set of 91 million variants derived from WGS of 722 individuals to identify genomic changes resulting from selective pressure occurring during breed formation and maintenance. The variant catalog produced here is comprehensive and includes data from wild canids, indigenous and village dog populations, and 144 domestic dog breeds. We hypothesize that unbiased analysis of variant allele frequencies will reveal genomic signatures of artificial selection for specific phenotypes^23^, and we therefore apply sequence-based GWAS to 16 breed traits using American Kennel Club standards as phenotypic measures^5,24^.

Leveraging our comprehensive sampling of 144 domesticated breeds, these analyses uncover a dozen newly associated genes, and in some cases, likely causative variants associated with morphological traits and life span. In this initial study, WGS data is used to directly perform GWAS for several canid traits. We next use WGS from wild canids and indigenous dogs in the catalog to refine our GWAS results, demonstrating that alleles which distinguish common breed-associated traits have been under selection since early breed formation. The work presented here demonstrates the utility of canine WGS data in expanding our genetic understanding of morphologic variation and its origins.

## Results

### WGS catalog

To comprehensively represent the diversity of modern canids, we obtained publicly available WGS data from the genera *Canis*, *Cuon*, and *Lycalopex* (Sequence Read Archive: http://www.ncbi.nlm.nih.gov/sra; *n* = 314 unique individuals), as well as 128 unpublished genomes contributed by collaborators, 186 previously catalogued WGS^25^ and data from 94 domestic dogs sequenced by the Ostrander lab of which 52 were previously unpublished and now available on NCBI (accession number: PRJNA448733). All Biosample numbers for the 722 genomes are listed in the Supplementary Data 1 and the entire genome dataset can be found on NCBI. Long-term health status of most dogs is unknown. We applied standard QC methods to remove duplicate samples (see Methods) and validated the breed/species of each genome using a neighbor joining phylogeny comprising variant positions and data from Parker et al.^4^ (Supplementary Fig. 1). The final reference dataset contained 722 WGS from 144 established breeds, with 54 breeds represented by three or more dogs, 11 mixed breed samples, 26 samples of unknown breed status, 104 village and feral dogs from diverse locales, and 54 wild canids from six species (Supplementary Fig. 2a and Supplementary Data 1). The complete data set (vcf file containing 91 million variants and 722 genomes) is also available on NCBI (accession number: PRJNA448733).

To find genomic patterns enriched within breeds selected and maintained by human intervention, variants were called across all 722 individuals. The vast majority of the 91 million variants (including 17.3 million small (+/−24 bp) indel variants) observed are contained within intergenic regions (Supplementary Fig. 2c). Thirty-five percent of variants, including those in wild canids, are within introns or exons, 39% of exonic changes are non-synonymous and 7% are high impact variants as defined by both snpEFF^26^ and VEP^27^ (Supplementary Fig. 2c and Supplementary Table 1). The sequence depth for the 722 WGS ranged from 2.0x to 93.8x with a median of 18x (Supplementary Data 1). To optimize the dataset, we use previously published SNP chip data^11^, collected from a subset of the same individuals, to determine the minimum sequence depth required for confident genotype calls and opt to use a genome quality score (GQ) of 20 and an average sequence depth >10x (Supplementary Fig. 2b). We then define a primary reference dataset that retains only biallelic SNVs and small indels, for a total of 76.5 million variants (Supplementary Fig. 3). For the studies described here, we further refine the dataset, retaining only two males and two females from each modern breed, selecting those with the deepest sequence coverage. We also remove the genomes of village, mixed breed and dogs of unknown origin, but retain the genomes of wild canines in order to ascertain ancestral versus derived alleles, thus generating a working dataset of 268 modern breeds dogs and 54 wild canids. Finally, we use village dogs as an outlier group in order to identify genomic signatures of artificial selection in modern breeds.

### Morphological traits analyses

**Fig. 1:**
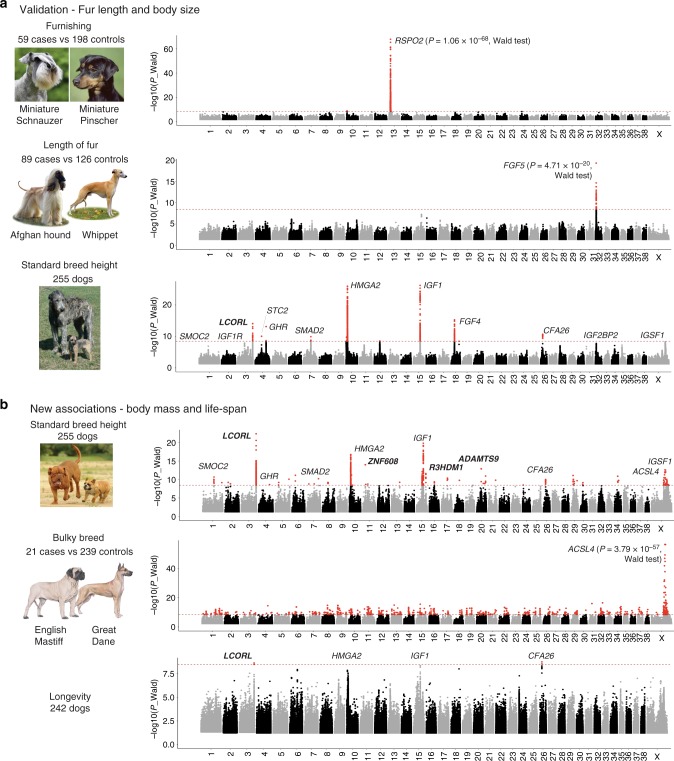
GWAS results for morphological traits in dogs using the canine 722 genome catalog. Manhattan plots showing statistical significance (−log10 scale) for the 30,000 most associated biallelic variants for each canine autosome, and all variants for the X chromosome (*X*-axis). **a** Validation of this WGS-GWAS approach using known examples in dogs: presence or absence of moustache and eyebrows, length of fur, and height as a multigenic trait. **b** Associations identified using body mass including the bulky phenotype and life span. The red line represents the Bonferroni corrected significance threshold (−log_10_(*P*) ≃8.46) and variants passing this threshold are colored in red. Candidate genes identified in this study are in bold

We investigated 16 phenotypes using a Genome-Wide Mixed Model Association algorithm (GEMMA)^28^ which fits a univariate linear mixed model for marker association tests with a single phenotype, correcting for sex and using a relatedness matrix to correct for population stratification (Supplementary Fig. 3). The number of breeds used for each analysis depends on the availability of the standard breed information of the American Kennel Club^5^ (Table 1 and Supplementary Data 2). Keeping only variants with minor allele frequency above 1%, genome-wide data from an average of 14 million variants per phenotype are analyzed. Bonferroni corrections are applied to identify significant associations (threshold = 8.46) (Tables 1 and 2). Our initial findings validate our previously described associations for Mendelian morphological traits including fur growth patterns^14^ and coat color^29^; as well as complex traits such as standard breed height (SBH)^1,13,16,18^ (Fig. 1a). The analysis for SBH highlighted only genes/loci previously described in dogs such as the *ligand-dependent nuclear receptor corepressor-like* gene (*LCORL*), *Stanniocalcin 2 (STC2), growth hormone receptor* (*GHR*), *SMAD family member 2 (SMAD2)*, *high mobility group AT-hook 2* (*HMGA2*), fibroblast growth factor 4 (*FGF4*), *insulin like growth factor 1* (*IGF1*), and one locus on *Canis lupus familiaris* chromosome 26 (CFA26)^1,13,16,18^. Signals at three previously identified genes, *insulin like growth factor 1 receptor (IGF1R), insulin like growth factor 2 mRNA binding protein 2 (IGF2BP2*) and *immunoglobulin superfamily member 1 (IGSF1)* were observed but did not pass the Bonferroni threshold (Fig. 1a).

**Table 1:** Summary of phenotypes used to perform GWAS using the WGS catalog

Phenotype	Number dogs	Number cases/controls	Number variants	Best *P* value	Bonferroni threshold: (−log 10 (0.05/Nb.variants))	Nb. Variants passing Bonferroni threshold
Canids catalog	722	—	76854926	—	—	—
Kinship	268	—	14489676	—	—	—
Aggressiveness	63	—	13191759	1.27E-07		0
Boldness	65	29/36	13545961	1.01E-10	8.42	21
Bulky	257	21/239	14437654	3.79E-57	8.43	1797
Drop ears	214	113/101	14426181	7.63E-24	8.46	1100
Furnishing	257	59/198	14387349	1.06E-68	8.46	976
Hairless	268	6/262	14489548	3.16E-67	8.46	3908
Height	255	—	14416697	6.35E-27	8.46	1074
Large ears	213	31/182	14457478	4.91E-41	8.46	1242
Lengh of fur	215	89/126	14352965	4.71E-20	8.46	43
Life span	242	—	14670938	1.77E-09	8.46	4
Long legs	102	22/80	13732336	6.24E-14	8.44	569
Muscled	244	52/192	14809625	2.69E-15	8.46	1175
Tail curl	173	—	14637750	4.47E-11	8.47	461
Weight	255	—	14416697	4.04E-23	8.46	938
White chest	195	100/195	14847812	3.75E-19	8.47	50
White head	179	57/122	14386917	5.99E-29	8.46	94

**Table 2:** Summary of significant associations identified by multiple GWAS using a maximum of 268 modern breed genomes

Position/region	*P* value	Gene/locus	Function	Associated phenotype in this study
chr1:42085782-42573240	2.31E-11	***ESR1***	Major mediator of estrogen action for bone mass/osteoporosis^42^	**Height (long legs)**
chr3:55954929-56065637	1.62E-10	***ADAMTSL3*** **locus**	Human, pig and cattle loan weight QTL^33^	**Bulky breeds**
chr3:91269525	4.04E-23	LCORL*	Transcription factor - body size^35–38^	Height, weight^18^, **life-span**
chr4:39182836	8.93E-11	*STC2*	Glycoprotein hormone - body size	Height^16,18^
chr4:66902902-67093815	7.51E-14	*GHR*	Growth hormone - body size	Height^16,18^
chr7:26603745-28240043	4.47E-11	***GORAB***	Gene involved in bones morphology^74^	**Curl tail**
chr7:43724293-43890274	1.26E-10	*SMAD2*	Transcription factor - body size	Height^16,18^
chr9:27659585	3.03E-40	***CA10***	Enzyme associated with metabolic syndrome^75^	**Hairless**
chr10:8070103	7.63E-24	***WIF1-MSRB3-lncRNA*** *	Multi-traits locus in dogs	**Drop ears** ^7, 52^
chr10:8351907-8488300	1.17E-26	*HMGA2*	Transcription factor - body size	Height^16,18^, boldness^7,52^
chr11:14030600	9.53E-15	***ZNF608*** **locus**	Body mass QTL in pig/body mass index QTL in human^30^	**Weight, Bulky breed**
chr11:18621251-18855024	2.39E-09	***CHSY3***	Associated with mechanical function in cartilage^76^	**Curl tail**
chr12:33803314-35061155	4.91E-41	***RIMS1-KCNQ5***	Neurological genes (cognition/potassium channel in cochlea)^47,48^	**Large ears**
chr13:8610419	1.06E-68	*RSPO2*	Fur length and furnishing gene	Furnishing^14^
chr15:41221438	6.35E-27	*IGF1*	Growth hormone - body size	Height, weight^13,16,18^, **life-span**
chr17:37651314	3.16E-67	*FOXI3*	Hairless in dogs	Hairless^77^
chr18:20447435	5.95E-16	*FGF4* retrotransposon	Chondrodysplasia	Height^18^
chr19:38303408	2.17E-13	R3HDM1*	Meat QTL in cattle^31^	**Weight, bulky breed**
chr20:21786368-21869849	5.99E-29	*MITF*	Coat color	White chest, white head^29^
chr20:26692625	1.21E-13	ADAMTS9-AS*	Human adiposity locus^32^	**Weight**
chr24:31856245	1.27E-07	***R3HDML***	Associated with psychotic illness in human patients^78^	**Aggressiveness**
chr26:12796099-13004170	2.08E-11	***TBX3-MED13L-RNFT2***	Body size locus	**Height, weight** ^18^ **, life-span**
chr29:23802662	1.95E-10	HNF4G*	Intramuscular fat deposition of beef cattle^34^	**Bulky breed**
chr32:4476417	4.71E-20	*FGF5*	Length of fur	Length of fur^14^
chr34:20097018-212633271	1.26E-11	*IGF2BP2* locus	Growth hormone - body size	Weight^18^
chrX:82919525	3.79E-57	*ACSL4*	Enzyme associated with body mass	Weight, bulky breed^17^
chrX:82310627-86057014	3.38E-13	**CFAX-locus 1 (** ***IRS4*** **)**	Body mass locus	Height, weight^17^, **life-span**
chrX:101732248-103320770	2.69E-15	**CFAX-locus 2** ***(IGSF1)***	Body mass locus	Height, weight, muscled breeds^17^, **life-span**

Bold indicates identification in this study, and asterisk denote previously unreported mutations

Region or exact positions are defined by variants passing the Bonferroni correction threshold (8.46)

We next run quantitative GWAS using breed-average measures for weight (SBW) as taken from the AKC breed standards (Fig. 1b). We identify 12 significant associations with weight (SBW) including the known canine body size genes/loci of *LCORL*, *GHR*, *SMAD2*, *HMGA2*, *IGF1*^16,18^, as well as the two recently described genes: *acyl-CoA synthetase long chain family member 4* (*ASCL4*) and *IGSF1*^17^ (Fig. 1b). Our analysis also reveals three candidate genes on CFA11 (*zinc finger protein 608-ZNF608*)^30^, CFA19 (*R3H domain-containing protein 1- R3HDM1*)^31^ and CFA20 (*ADAM metallopeptidase with thrombospondin type 1 motif 9 - ADAMTS9*)^32^. In addition, we identified two genes, *ADAMTS-like protein 3* (*ADAMTSL3*)^33^ on CFA3 and the *hepatocyte nuclear factor 4-gamma* gene (*HNF4G*)^34^ on CFA29 associated with the tall heavy muscled (bulky) phenotype we described previously^17^.

**Fig. 2:**
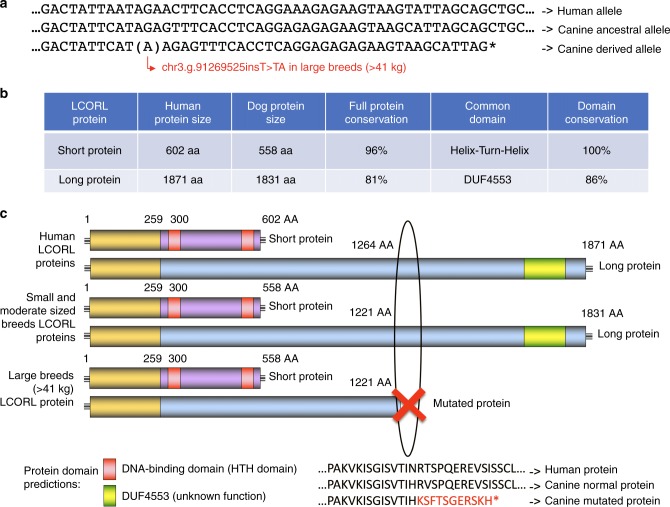
Identification of LCORL mutation in large breeds and comparison with human. **a** Comparison of genomic sequences between human and the two canine alleles. A single nucleotide insertion is observed in large breeds (>41 kg). **b** Conservation of the two main LCORL proteins and their predicted functional domain using SIM^68^ and LALNVIEW^69^. **c** Schematic representations of LCORL proteins, highlighting the effect of the canine mutation (STOP codon after amino acid 1221 leads to a loss of 610 aa). The common part shared by all forms is colored in yellow. Source data are provided as a Source Data file

We observe a significant association at *LCORL* in the analysis of both SBW and SBH (*p*_wald_ = 4.1 × 10^−23^ and 2.4 × 10^−10^, respectively), which are themselves highly correlated traits. No canine mutation has been previously described for this gene which encodes a transcription factor that has an established association with body size in other species^35–38^. The human gene has several isoforms, one of which is “long” (5,493 bp-NCBI: XP_022272118.1) and several that are “short” (≃1600 bp), differing significantly in the sequence of the last exons (4850 bp and 1301 bp, respectively) (Fig. 2b, c). Sanger sequencing of cDNA obtained from testis reveals three canine isoforms, two short and one long (Supplementary Data 3). Examination of both the WGS and testis cDNA reveals that large breeds (SBW > 41 kg) harbor a 1-bp insertion in the last exon of only the long isoform (Fig. 2a). With an allele frequency of 0.18 in the modern breed population, this mutation was never observed in small breeds (<10 kg), has a low frequency (af = 0.16) in medium sized breeds (between 10–41 kg), and is present in 80% of large breeds (>41 kg) (af = 0.67) (Supplementary Data 4). This insertion introduces a frameshift, changing the sequence of 11 amino acids and creating a premature stop codon (p.S1221*), resulting in the loss of 611 terminal amino acids (Fig. 2c). Alignment of human (ENSP00000490600.1) and canine LCORL protein sequences revealed strong conservation, with 81% identity. Interestingly, the long form of the protein contains a DUF4553 DNA-binding domain within the deleted portion of the dog protein. The strong conservation of this DNA-binding domain (86%) between human and dog suggests that, in large dogs, the 611 amino acid loss may disrupt transcription factor binding of LCORL with its target.

In addition to the above, regulatory element variants associated with canine SBW are identified in *R3HDM1*, *ADAMTS9* and *HNF4G*, affecting promoter, long non-coding RNA and 3’UTR, respectively (Table 3). As expected, the identified body weight variants were never or rarely observed in wild canids (af < 0.06), defining them as derived alleles (Supplementary Data 4). Presence of the derived alleles in wild canids with low allele frequencies can be explained by post-divergence gene flow between wild canids and dog populations, and has been previously reported^8^. We also observe lower allele frequencies in village dogs compared to modern breeds, reflecting the absence of selective pressure in village dog populations for the specified body size genes under selection in modern breeds. The single exception was an allele frequency of 0.17 in wild canids and 0.59 in village dogs for the derived allele of *IGSF1*, which has been previously associated with the muscled phenotype in domestic dogs^17^, perhaps providing a fitness advantage in the “village dog” environment.

**Table 3:** Previously unreported candidate variants identified using the WGS canids catalog

Associated phenotype	Gene/lncRNA	Definition/function	*P* value (Wald test)	Locus/position	Best candidate variation(s)
Height (long legs)	*ESR1*	Estrogen receptor 1	2.31E-11	chr1:42085782-42573240	Intronic SNPs
Height, weight	*LCORL* ^a^	Ligand-dependent nuclear receptor corepressor like	4.04E-23	chr3:91269525	Indel in the last exon - lead to a STOP codon
Drop ears	*TCONS_00016758* ^a^ *TCONS_00016759* ^a^	Mutated lncRNA 29 kb downstream the last exon of *MSRB3*	7.63E-24	chr10:8070103	Exonic SNP in one lincRNA
Weight	*ZNF608 locus*	Zinc finger protein 608	9.53E-15	chr11:13906259-14081398	SNPs 200 kb downstream the last exon
Weight, bulky	*R3HDM1* ^a^	R3H domain containing 1	2.17E-13	chr19:38303408	CpG island - promoter
Weight	*ADAMTS9-AS* ^a^	ADAM metallopeptidase with thrombospondin type 1 motif 9	1.21E-13	chr20:26692625	exonic variant
Bulky breed	*HNF4G* ^a^	Hepatocyte nuclear factor 4 gamma	1.95E-10	chr29:23802662	3’UTR SNP

^a^Mutated transcript

We confirm all body size variants by Sanger sequencing DNA from 468 independent dogs encompassing 96 breeds of varying size and shape (five dogs/breed minimum) (Supplementary Data 5). We observed low allele frequencies (<0.03) for the described mutations in *R3HDM1*, *ADAMTS9* and *HNF4G*, as estimated with the WGS data set. The derived allele for each of these genes was only observed in bulky breeds, including the Bernese Mountain Dog, Great Dane, English Mastiff, and Saint Bernard (Supplementary Data 4 and 5).

Combining our results with previously published data^13,15–17^, we estimate that variants in just 14 genes, *i.e. IGF1R*, *LCORL*, *STC2*, *GHR(1)*, *GHR(2)*, *SMAD2*, *HMGA2*, *ZNF608*, *IGF1*, *R3HDM1*, *ADAMTS9-AS*, *HNF4G*, *ACSL4*, and *IGSF1* account for as much as 95% of SBW variation in purebred dogs (Table 4). Thus, while several hundred loci affect human height and body mass index (BMI)^33,38,39^, a much smaller number of genes of large effect explain the striking 40-fold range of body size observed across dog breeds.

**Table 4:** Allele frequencies at 14 markers explain 95% of weight variation in dog population

Gene/locus	Mean SBW of D/D dogs (in kg)	Position	Modern dog breeds population			Small breeds (<10 kg)	Medium breeds (10 < SBW < 41 kg)	Large breeds (>41 kg)	Wild canid	Village dogs
			Af	beta	Variance	Af	Af	Af	Af	Af
*LCORL*	*43.9* ± *13.6*	chr3:91269525	0.17	11.15	0.159	0.00	0.01	0.68	0.00	0.10
*IGF1*	*13* ± *10.5*	chr15:41221438	0.44	−7.72	0.131	0.85	0.35	0.18	0.00	0.19
*HMGA2*	*6.3* ± *3.1*	chr10:8351907	0.21	−8.92	0.116	0.87	0.03	0.05	0.06	0.08
*ACSL4*	*50.3* ± *12.5*	chrX:82919525	0.08	12.50	0.100	0.00	0.03	0.55	0.00	0.00
*ZNF608*	*70.3* ± *0.1*	chr11:13945821	0.02	27.33	0.118	0.00	0.00	0.16	0.00	0.00
*ADAMTS9-AS*	*66.3* ± *4.6*	chr20:26661051	0.02	21.46	0.096	0.00	0.00	0.21	0.00	0.00
*R3HDM1*	*67.3* ± *5.2*	chr19:38303408	0.01	14.55	0.019	0.00	0.00	0.18	0.00	0.00
*SMAD2*	*9.1* ± *6.4*	chr7:43782633	0.24	−5.95	0.057	0.57	0.16	0.05	0.01	0.03
*GHR (1)*	*10.1* ± *7.4*	chr4:67040898	0.23	−4.53	0.032	0.68	0.19	0.04	0.00	0.07
*IGSF1*	*32.5* ± *17*	chrX:102292529	0.38	4.63	0.045	0.19	0.34	0.96	0.17	0.59
*STC2*	*8.2* ± *5.5*	chr4:39182836	0.18	−5.29	0.036	0.47	0.10	0.11	0.00	0.05
*HNF4G*	*52.2* ± *15.7*	chr29:23802662	0.01	9.97	0.009	0.00	0.00	0.20	0.00	0.01
*IGF1R*	*3.25* ± *0.1*	chr3:41849479	0.04	−7.70	0.018	0.13	0.01	0.00	0.00	0.01
*GHR (2)*	*7.03* ± *0.1*	chr4:67040939	0.01	−10.17	0.013	0.06	0.00	0.00	0.00	0.00
Af: allele frequency for the derived allele				Total	0.95					

Beta: estimated SNP effect (regression coefficient)

Derived allele is the allele absent (or present with a low frequency) into the wild canid population. The genetic variance attributable to each variant was estimated as V = 2Af(1 − Af) × beta2/variance(SBW)

In order to provide more information about functional impact of these genes on body size, we utilized 51 RNA-seq experiments from SRA database and, in parallel, isolated RNA from 28 testes from 20 breeds for qRT-PCR analysis (Supplementary Data 6 and 7). As expected, we do not observe significant differences in either analysis, as the number of breeds is low and, in many cases, ideal tissue types were not available (Supplementary Fig. 4 and Supplementary Data 6 and 7).

### Longevity analysis

**Fig. 3:**
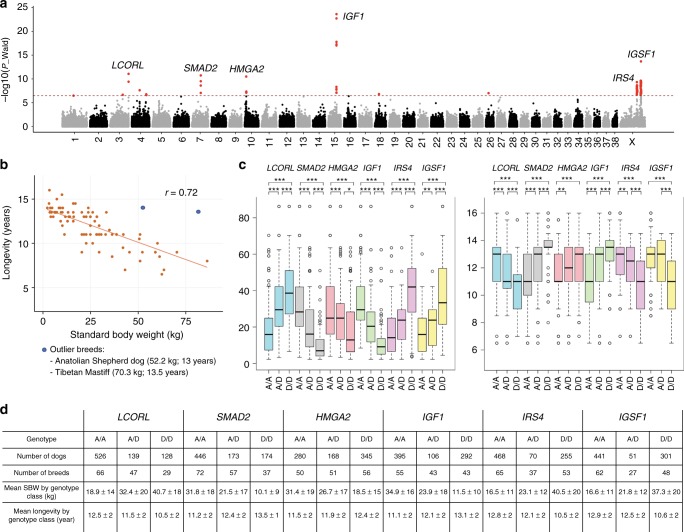
Body mass and longevity analyses using 746 dogs genotyped on 170k SNP markers. **a** Manhattan plot of the multivariate GWAS for standard breed weight (SBW) and life span corrected by sex, using 746 dogs genotyped on Illumina HD SNP array^11^. The −log10 *P* values for each SNP are plotted on the *y*-axis versus each canine autosome and the X-chromosome on the *x*-axis. The red line represents the Bonferroni corrected significance threshold (−log_10_(*P*) = 6.48) and SNPs passing this threshold are colored in red. **b** Negative correlation between SBW and longevity. In blue, large breed outliers: Anatolian Shepherd Dogs (52.2 kg; 13 years) and Tibetan Mastiff (70.3 kg; 13.5 years) **c** SBW and longevity (*y*-axis) of each breed (without outliers) are plotted by genotype at each marker (*x*-axis). The homozygous D/D alleles have generally a stronger effect on the distribution of SBWs (or longevity) for a given genotype/marker combination (the median and first and third quartiles are indicated by the box-plots). Statistics for each genotype/marker combination are summarized in (**d**). *P* values estimated by Mann–Whitney–Wilcoxon tests (*P < 0.05; **P < 0.01; ***P < 0.001). SBWs and longevity of genotype classes are reported as mean ± SD. Source data are provided as a Source Data file

We next considered the role of genetic predisposition in life span using American Kennel Club (AKC) breed-average life spans as a phenotype. Four of the 17 body weight/size loci identified in this study are significantly associated with longevity: *LCORL*, *HMGA2*, *IGF1* and the locus on CFA26 (Fig. 1b). These results support and partially explain the previously reported correlation between body size and life span in domestic dog; large breeds breeds (SBW >30 kg) have a shorter average life span (8–10 years) than miniature and toy breeds, which can live ≥ 18 years^24,40^. We further investigate this observation using a panel of 746 dogs from 79 breeds genotyped using the Illumina Canine HD SNP array^11^ (Supplementary Data 8). Using the AKC metrics of breed-average for both weight and life span^5^, we observe a negative correlation between these traits (*r* = 0.72) (Fig. 3b). We use GEMMA^28,41^ to perform an association test with multivariate linear mixed models, which simultaneously estimates the association between a given variant and phenotypes of interest^41^, in this case body size and breed average lifespan (Fig. 3a and Supplementary Fig. 5), and observed the most significant associations (*p*_wald_ < 5 × 10^−10^) for *HMGA2*, *IGF1*, *IGSF1*, *IRS4*, *LCORL* and *SMAD2*.

We test which genes contribute the most to both body size and life span, defining the “ancestral” allele for each gene (as opposed to “derived”) as that present in wild canid genomes (Supplementary Data 4). For *SMAD2*, *HMGA2* and *IGF1*, the derived allele is associated with low SBW (average = 12.7 kg) and increased longevity (avg = 13 years), (*p* < 0.001, Mann–Whitney– Wilcoxon test). An increase in SBW and reduced lifespan (avg SBW = 39.5 kg; avg life span = 10.5 years; *p* < 0.001, Mann–Whitney–Wilcoxon test) are also observed in breeds homozygous for the derived allele of the most strongly associated marker at *LCORL*, *IRS4* and *IGSF1* (*p* < 0.01, Mann–Whitney–Wilcoxon test). Finally, a reduced life span is observed only for those breeds homozygous for the derived allele at *IGSF1* (avg = 10.6, *p* < 0.001, Mann–Whitney–Wilcoxon test).

### Additional morphologic phenotypes

**Fig. 4:**
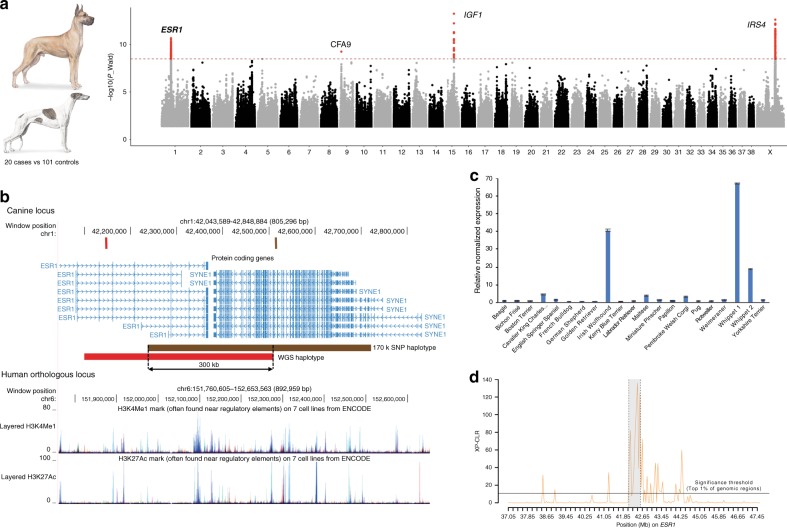
*ESR1* and the long leg phenotype in dogs. **a** Manhattan plots showing statistical significance (−log10 scale) for the 30,000 most associated biallelic variants for each canine autosome, and all variants for the X chromosome (*X*-axis) for the long-leg phenotype observed in Sighthounds, Great Dane, and Great Pyrenees. We distinguish four peaks: one peak pinpointing *ESR1* gene on chromosome 1, one locus on CFA9 without any candidate genes in the interval, and *IGF1* (CFA15) and *IRS4* (CFAX) previously associated with height variation in dogs. Images to the left are Great Dane (top) and Greyhound (bottom). **b** UCSC genome browser showing the *ESR1* locus in dog (top) and human (bottom). Vertical bars correspond to the most associated variants identified with the 722 genomes (in red), and the 855 dogs genotyped on 170k SNP array (in brown), and horizontal bars represent the homozygous haplotype observed. The bottom panel represents the human orthologous locus with tracks corresponding to the H3K4me1 and H3K27ac chromatin signals annotated by the ENCODE project^55^. **c** Expression level of *ESR1* in a panel of 20 breeds, showing high expression in the Sighthounds, Irish Wolfhound and Whippet, in comparison to six different breeds with average leg length. *Y*-axis represents the relative normalized expression. **d** XP-CLR plot on *ESR1* locus comparing Sighthounds (long legs breeds) with normal-sized legs breeds. We detected a significant selection signature located on *ESR1* locus (in grey). Horizontal lines represent the empirical top 1% of genomic regions. Source data are provided as a Source Data file

We investigate several additional morphologic phenotypes including leg length, ear shape, and tail length and curl. We compare 22 dogs from 10 breeds with long hindquarters, as defined by the AKC^5^, including Sighthounds and tall working breeds (*i.e*. Great Dane, and Great Pyrenees (Fig. 4a)) versus 48 other breeds (80 small, medium and large dogs) and we find four large homozygous haplotypes that are significantly associated with long legs. The first and second, spanning the *IGF1* and *IRS4* genes have been previously described as body size genes^13,17^, and are validated herein (*IGF1*: *p*_wald_ < 6.2 × 10^−14^ and *IRS4*: *p*_wald_ < 2.6 × 10^−13^). Two associations on CFA1 (42–42.5 Mb) and CFA9 (53.4–54 Mb) were also observed. While no genes are annotated for the interval on CFA9, the association observed on CFA1 spans the *estrogen receptor 1* (*ESR1*) gene, with the most significant variant located within the second intron of the gene (Fig. 4b). *ESR1* is a major mediator of estrogen action, and is strongly linked to bone mass and osteoporosis in humans^42^. We confirm the CFA1 locus association using 855 dogs (88 breeds) genotyped on the Illumina Canine HD SNP array (Supplementary Table 2) and observe that >80% of long-legged dogs harbor the derived allele for the most associated SNP. Combining haplotype data from the 102 WGS and 855 genotyped dogs, we reduce the locus to 300 kb, spanning both *ESR1* and its neighboring gene *Spectrin Repeat Containing Nuclear Envelope Protein 1* (*SYNE1*). No mutations were identified within exonic sequences of either gene. However, examination of the human orthologous region reveals numerous annotated histone marks on the locus suggesting non-coding variants modulating regulatory elements in long-limbed dogs (Fig. 4b). qRT-PCR analysis using RNA extracted from testes revealed significantly higher levels of *ESR1* expression in Sighthounds, with Irish Wolfhounds and Whippets displaying 20–70 times higher levels of *ESR1* than other tested breeds (Fig. 4c). These results suggest that either over-expression of *ESR1* is involved in a process leading to the elongation of long bones and epiphyseal fusion^42^, and/or that variation in gene expression is associated with an ossification disorder. The latter is of particular interest as many long-legged breeds are predisposed to develop bone diseases, including osteosarcoma^21^, for which *ESR1* is reportedly a contributing factor^43^.

**Fig. 5:**
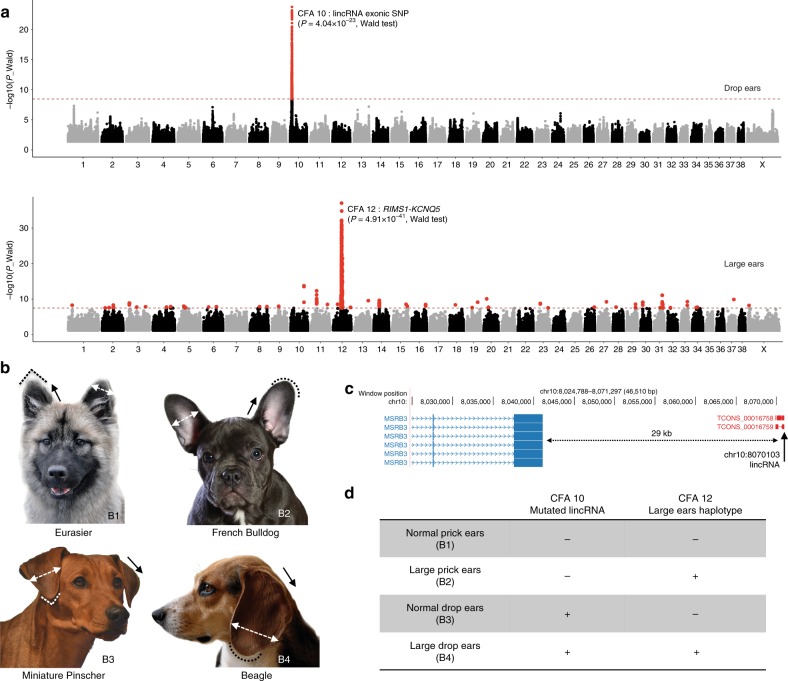
Ear morphology in dogs. **a** Manhattan plots showing one significant signal on the CFA10 for the drops ears phenotype and another one on chromosome 12 for the large and round ears. **b** Characteristic breeds representing four different ear shapes observed in dogs: Normal (1,3), large and round (2,4), prick (1,2) or drop (3,4). **c** UCSC genome browser showing the position on the canine genome (Canfam3.1) of the mutated lincRNA (in red) associated with the drop ears. **d** Combination of alleles at both loci create four phenotypes. Plus (+) and minus signs (−) indicate the presence or absence of variant (non-ancestral) genotype

We next sought genes underlying ear shape and size. The shape of the auricular cartilage determines the appearance of the pinna, which may be upright (prick ears) or pendulous (drop ears)^44^ (Fig. 5b). We compare variants from 60 breeds (113 dogs) with drop and 46 (101 dogs) with prick ears (Fig. 5a), and identify a significant association on CFA10 (*p*_wald_ = 7.63 × 10^−24^) with a single nucleotide variant (chr10.g.8070103C > T) located in the exonic region of a long intergenic non-coding RNA (lincRNAs) (*TCONS_00016758, TCONS_00016759*) (Fig. 5c). This lincRNA is 29 kb downstream from the gene *methionine sulfoxide reductase B3* (*MSRB3*), which is associated with human deafness^45,46^ (Fig. 5c and Table 3). The derived allele is detected in 76% of the drop ears dogs present in the WGS catalog, while only 5% of the prick ears dogs and wild canids carry the derived allele (Supplementary Data 4). Sanger sequencing of 855 dogs (88 breeds) reveals similar proportions, as 71 and 8% of drop and prick ear dogs carry the derived allele, respectively (Supplementary Table 3). Since the variant impacts a lncRNA, we hypothesize that a complex regulatory mechanism may be involved in determination of the drop ear phenotype, which includes this lincRNA, directly or indirectly, impacting *MSRB3* expression.

We also perform GWAS to identify genes controlling large, round ears (*e.g*. Spaniel breeds, Beagle and Corgi) versus triangular, standard size ears (*e.g*. Eurasier or Miniature Pinscher) (Fig. 5b). Large ears are defined as having a greater area between the lateral and medial border of the ear, with a round and not triangular apex^44^. Comparing WGS from 31 dogs of 13 breeds with large, round ears to 182 dogs (85 breeds) that lack this phenotype we observe a significant association on CFA12 (*p*_wald_ = 4.91 × 10^−41^). Analysis of variants either homozygous or heterozygous for a derived allele defined an interval of 33.8–35.1 Mb (Fig. 5a) which contains two genes: *Regulating Synaptic Membrane Exocytosis 1 (RIMS1)*, a gene involved in cognition processes in humans^47^, which is an unlikely candidate, and *Potassium Voltage-Gated Channel Subfamily Q Member 5* (*KCNQ5*). The latter has a vestibular role in mouse models^48^ and is a much stronger candidate. We did not detect coding variants in either gene, leading us to postulate non-exonic SNVs or structural variants as potential candidates involved in this phenotype. Acquisition of cartilage tissue, which has proven difficult to obtain, will allow future expression studies for both phenotypes (Supplementary Data 6 and 7, and Supplementary Fig. 6). Nevertheless, it is clear that combinations of variants at just these two loci control otherwise seemingly complex ear phenotypes in modern breeds (Fig. 5d). Other phenotypes (hairless, tail shape, behaviors) are described in Supplementary Fig. 7.

### Signatures of selection on candidate genes

To further substantiate our hypothesis that genes responsible for the marked phenotypic variations among dog breeds have been driven by positive selection, we use the cross-population composite likelihood ratio (XP-CLR)^49^ and cross-population extended haplotype homozygosity (XP-EHH)^50^ to investigate extreme allele frequency and LD differentiation over extended linked regions in multiple breeds. Hypothesizing that breeds with different traits have experienced distinct evolutionary processes, we performed five independent case/control analyses based on a subset of traits previously defined: (1) long legs; (2) bulky (tall heavy muscled); (3) standard breed height/weight; (4) drop ears, and (5) large ears (Table 5 and Supplementary Data 9–11) with a goal of localizing signals of population-specific selection. Using the empirical top 1% of genomic regions, most of the candidate genes (13 of 18) identified from GWAS show significant allele frequency, or LD differentiation, between case and control populations (Table 5 and Supplementary Fig. 8), suggesting that human selection caused adaptive mutations to sweep to high prevalence or become rapidly fixed within a population. Nine of 13 significant genes are detected by both tests. The *ESR1* gene, for example, reveals significant signals of positive selection (XP-CLR = 109.0, XP-EHH = 1.16) in breeds from the Sighthound clade (described in Supplementary Data 10 and 11) compared to average and short-legged breeds (Fig. 4d). We apply the same strategy to comparisons of case population versus random-bred village dogs and find that selection signatures remain significant (16 of 18 under a more relaxed threshold of 5%), highlighting the robustness of our results (Supplementary Data 10 and 11). Finally, the genetic distance between breeds of large and small size is significantly greater when estimated within body size genes compared to the whole genome (*P* < 2.2 × 10^−16^, Mann–Whitney U-test), based on the fixation index (F_ST_) (Supplementary Fig. 9).

**Table 5:** Summary of XP-CLR and XP-EHH analyses between domestic dog breeds

Analysis	Gene	XP-CLR	XP-EHH (*P* value^a^)
Long legs (Sighthounds)	*ESR1*	109.0^b^	1.16 (0.005)
Bulky	*ADAMTSL3* locus	7.3^c^	0.94 (0.012)
	*ZNF608* locus	22.5^b^	1.02 (0.0054)
	*R3HDM1*	NS	1.02 (0.0056)
	*HNF4G*	4.7^c^	NS
	*ADAMTS9-AS*	3.3^c^	NS
	*ACSL4*	NS	NS
	CFAX-locus 1	197.9^b^	1.66^b^
	CFAX-locus 2 *(IGSF1)*	110.1^b^	2.18^b^
Height/weight (small breeds)	*STC2*	7.19^c^	1.99 (0.000039)
	*GHR*	55.6^b^	1.17 (0.0025)
	*SMAD2*	5.0^c^	0.84 (0.04)
	*HMGA2*	173.7^b^	NS
	*IGF1*	169.3^b^	NS
Height/weight (large breeds)	*LCORL*	14.6^b^	1.91 (0.0002)
	*IGF2BP2* locus	21.3^b^	1.37 (0.0025)
Drop ears	*WIF1-MSRB3-lncRNA*	224.3^b^	1.49 (0.00025)
Large ears	*RIMS1-KCNQ5*	452.6^b^	1.78 (0.00012)

X chromosome was separated from autosomes to define the empirical top 1% of regions for both XP-CLR and XP-EHH. *P* values were not assigned for genes in the X chromosome (ACSL4, CFAX1, CFAX2)

*NS* non significant

^a^Rank-based empirical *P* value

^b^Significant under 1% threshold

^c^Significant under 5% threshold

## Discussion

We have generated an expansive catalog of canine genomic variation, identifying 91 million variants in 722 WGS. Using WGS from 268 canines, and analyzing over 76.8 million biallelic variants, we identified variants associated with common phenotypes observed across modern dog breeds but absent in wild canids. In total, 28 significant associations were detected, previously identified loci were validated, and a dozen previously unidentified genes and five mutations were found to be strongly associated with the traits tested (Tables 2 and 3). The approach differs significantly from previously published studies of genetic associations, which have relied on association tests using small to modest numbers of SNPs and, more recently imputation, to analyze a single phenotype. In those studies, WGS data or targeted sequencing was used to identify candidate variants^3,6,17,21,25,51,52^. The primary challenge of that approach is the multi-Mb LD observed in dog genomes^10–12^, resulting in a frequent inability to move from associated marker to genes/mutations^1,16,18,21^, thus limiting the utility of the dog for genetic studies. Recently, Broeckx et al. attempted to overcome this problem by comparing whole exome sequencing (WES) to SNP genotyping in a small number of dogs^53^. Using simulated phenotypes they showed, as expected, that WES-based GWAS has higher power than moderately dense (220 K) SNP chips to detect associations. However, while the approach is useful for finding exome based mutations^54^, it misses most regulatory mutations, which is where many high impact variants are likely to be^55^.

The canine data set produced here increases the catalogue of available genetic variants from thousands to 91 million. It is comprehensive, containing information on 144 domestic breeds, thus providing a robust dataset for identifying functional variants associated with morphologic traits, disease risk and behavior. In addition, the inclusion of wild canids and indigenous dog genomes provides an efficient mechanism for identifying ancestral versus derived alleles at any locus and, thus, studies of domestication. However, the lack of phenotypes for village dogs is a limitation when interpreting this data, hence we recommend a thoughtful application of village dog data in future investigations.

In this study we utilize breed standard measurements as phenotypes. This approach has been well-studied^24^ and validated^1,16–18^, as breed registries set stringent criteria for the appearance of each breed. This approach is thus the norm for mapping breed-associated traits. Collection of individual measurements would be needed to apply this dataset to mapping of within-breed variation.

This study greatly enhances understanding of canine body size genetics. The largest and smallest breeds differ in size by nearly 40-fold^5^. Yet within each breed male and female height are often specified to within one to two inches and mass to within a few kilograms^5^. Previous studies identified 17 QTLs associated with body size including both weight and height variation^1,16–18^. We previously showed that *GHR*, *HMGA2*, *IGF1*, *IGF1R*, *SMAD2* and *STC2* genes accounted for 64.3% of size variance in breeds with a SBW ≤ 41 kg (90 lb)^16^, but relatively little for breeds >41 kg. We also demonstrated that 90% of dogs weighing ≥41 kg share the same 2 Mb chromosome haplotypes for *IRS4, ACSL4* and *IGSF1*, which contributes to the bulky versus lean appearance of tall dogs^17^. In this study, we advance those results, identifing eight body size loci. Four genes (*ADAMTS9*, *HNF4G*, *R3HDM1*, *ZNF608*), together with a subset of previously reported genes (*IGSF1*, *GHR*, *SMAD2*, *STC2*) make small contributions to SBW variance, accounting for ≈2–9%. By comparison, *HMGA2*, *IGF1*, and *LCORL* each account for ≈12–15% of variance.

In this study, the density of WGS generated variants allows us to bypass fine mapping steps and directly identify likely functional mutations for four body size genes: *LCORL*, *R3HDM1*, *ADAMTS9-AS*, and *HNF4G*. In large dogs, we uniquely observe a single base insertion in *LCORL* that causes a premature stop, truncating 600 bp of a long isoform of the protein (Fig. 2). The *LCORL* locus has been previously associated with body size variation in dogs^7,18^, humans, cattle, pigs and horses^35–39^. Interestingly, in cattle and horses the most significantly associated SNPs are in a region that aligns with the last exon of the canine long isoform that is not yet annotated for the above domesticated species^36^. Combining our results with human and livestock data, we propose that variation in the *LCORL* accounts for a large proportion of body size variation in not only dogs, but most other mammals as well.

When the data presented here is combined with existing information^1,13,15–18^, we conclude that most body size variation in domestic dogs is likely accounted for. We readily acknowledge, however, that our prediction of 95% will change with the inclusion of more rare and unusual breeds, as is currently underway. Recently, a meta-analysis GWAS comparing data from over 58,000 cattle WGS identified 163 body size loci, revealing common body sizes genes shared with dogs and humans^35^. This is perhaps not surprising as large GWAS have identified hundreds of loci contributing to human body mass index (BMI), weight and height^33,38,39^. While other body size genes surely remain to be found in dogs, the final number is unlikely to approach that observed in human. This observation reflects the recent domestication of dogs, *i.e*. most breeds have existed for <250 years^1–4^ and result from strong selective pressure leading to rapid breed development.

Our canine body size studies dovetail well with those of breed longevity. In this study, genes underlying both body size and longevity have been investigated with high sensitivity in term of SNPs density and sample size. While previous studies have highlighted the observation that small dog breeds live, on average, longer than larger breeds^24,40^, this data demonstrates clearly that only a subset of body size genes, *i.e. HMGA2*, *IGF1*, *IGSF1*, *IRS4*, *LCORL* and *SMAD2*, are specifically related to life span. This sets the stage for more detailed within-breed experiments.

Exploration of additional morphological features using GWAS allows us to identify genes such as *ESR1*, which is associated with long legs, a mutated lincRNA downstream of *MSRB3* associated with drop ears, and *KCNQ5* which is associated with large and round ears. All of the associated genes have biologically plausible links to the associated traits, although precise bone measurements from X-rays would allow us to extend our studies^56^. Among the most interesting genes are those associated with ear shape. *KCNQ5* is a member of the K^+^ channel family is strongly associated with hearing in mice^48^. No published studies demonstrate differential expression of *KCNQ5* in dogs with large versus normal-sized ears, nor differences in hearing ability, suggesting multiple as yet unrecognized functions for the gene, or alternative roles in the presence of gene mutations. When considering the drop-ear phenotype, the most likely explanation is cis-repression or activation of neighboring genes caused by changes in an adjacent lincRNA on CFA10^57^.

Previous studies have highlighted CFA10, on which *MSRB3* is located, as associated with ear morphology^7,52^ (Supplementary Fig. 7), but no gene or causal mutation has been reported to date. We propose that either *MSRB3*, an adjacent gene associated with human deafness^45,46^ or *Wnt inhibitor factor 1* (*WIF1*), which is located 140 kb upstream from *MSRB3* and is associated with ear morphology in pigs^58^, may be the target of the mutated lincRNA. Previous studies comparing pigs who had drop versus prick ears did not reveal obvious high impact mutations in either gene, but did demonstrate higher *WIF1* and lower *MSRB3* protein expression in prick versus non prick eared pigs^58^. In aggregate, these results may suggest that coordinated expression of both *MSRB3* and *WIF1* is important for ear shape. As with body size genes, the observations associated with ear morphology highlight a recurring theme in dog genetics; i.e., that small numbers of genes/RNAs control seemingly complex phenotypes (Fig. 5). The recent publication of an annotation of missing exons and lincRNA in the dog genome highlights needed studies that will facilitate future explorations aimed at finding causative mutations in the dog^59^.

All genes identified in this study likely exemplify the myriad evolutionary processes that have shaped phenotypic variations of modern dog breeds and, accordingly, were further evaluated for signatures of selection. In an effort to pinpoint the true signal, we combine both independent selection scans (XP-CLR and XP-EHH), and find that most of the candidate genes reveal significantly long haplotypes of population differentiation between case and control populations, as defined by breed standard phenotype. We further compare each case population to the random-bred village dogs, which have not undergone structured breeding, and observe that modern breeds have experienced different levels of selective pressures to obtain the desired phenotypes. These results together suggest that the observed mutations are unlikely to have been the result of random genetic drift, rather they result from positive selection which impacted the genetic landscape of mutations within and across diverse clades. Also, given that this study assigns multiple breeds to a single case phenotype (*i.e*. multi-breed approach), it is possible that additional genes with little to no evidence of selection may have contributed to breed-specific trait variation. It is worth noting that the incidence of false positives can be further minimized by taking into account the inferred demographic model and parameters of each modern breed with advances in our understanding of complex demographic history.

The diversity and number of breeds, village dogs and wild canids in the dataset ensures that much, if not most, of the genetic variation present in modern canids, 18% of which are indels and 82% which are SNVs, are captured in this study. This will facilitate the identification of breed-specific and shared genomic variation, including that associated with complex diseases. As the number of canids in the catalog increases, so will its power. The current dataset, for instance, does not yet include large structural variants and the catalogue is Euro-American centric, particularly lacking breeds from Asia and Africa. This will be remedied in the near future by the inclusion of data from the international Dog10K project (dog10kgenomes.org), which is performing WGS on 10,000 canines representative of all continents in the next five years. In the immediate timeframe, the addition of the remaining AKC and Fédération Cynologique Internationale (fci.be/nomenclature/) breeds, particularly those from rare breeds and under-represented clades, will advance the utility of the catalog quickly. We encourage all investigators with WGS to make their data public for inclusion in future versions of the catalogue quickly. We particularly encourage the entry of registered dogs into the dataset as breed-specific metrics can be directly used as phenotypes. This study, then, provides a blueprint for expanding the utility of the canine system for identification of variants, genes, and pathways critical to mammalian health and biology.

## Methods

### Whole genome sequencing samples

WGS data utilized in this study was gathered from the Sequence Read Archive (SRA; http://www.ncbi.nlm.nih.gov/sra; *n* = 500 unique individuals), contributed by collaborators (*n* = 128) or generated by the NIH Intramural Sequencing Center (*n* = 94 total including 52 not previously published and now available on NCBI: accession number PRJNA448733). For the SRA data, domestic dog or wild canid data deposited in SRA prior to April 2017 were used in this study. All Biosample numbers for the 722 genomes are listed in the Supplementary Data 1 and the entire genome dataset can be found on NCBI [https://www.ncbi.nlm.nih.gov/bioproject/PRJNA448733]. After alignment and variant calling (see Supplementary Fig. 3 for the full description of the pipeline and references), samples were removed if they were low quality, *e.g*. less than 2x average depth, contained corrupt data (see “breeds and variants analyses” sections), or found to be duplicate individuals using the ‘genome’ function in plink version 1.9^60^. The final dataset consisted of 54 wild canids, 526 purebred dogs, and 142 random-bred dogs, and includes village and indigenous dogs, known mixes and dogs with unknown or uncertain heritage. The complete data set (VCF file containing 91 million variants and 722 genomes) is also available on NCBI [https://www.ncbi.nlm.nih.gov/bioproject/PRJNA448733].

### Whole genome sequencing and SNP chip concordance

To demonstrate the importance of genotype quality filters in genomes with lower average depth, concordance between WGS and Illumina Canine HD SNP chip genotypes were calculated as percent WGS “no call” genotypes. For the 722 genomes dataset, this is of particular importance as the average depth ranges from 2.0x to 93.8x with a median of 18x (Supplementary Data 1). A subset of forty genomes with >30x average depth and Illumina Canine HD SNP chip genotypes were utilized to identify a set of SNVs with 100% concordance between the WGS and 150,112 SNP chip genotypes, which were termed “high quality SNVs” in a previous analysis^4^. The SNP chip genotypes were converted to match the dog genome reference/alternate alleles (canfam3.1 assembly) at 145 loci using the plink-flip command. The file was converted to vcf format using plink-recode vcf and -a2-allele with a list of reference alleles for each locus from the canfam3.1 assembly^60^. Discordance was calculated using vcftools with the file comparison option–diff-site-discordance to identify the SNPs with 100% concordance and–diff-indv-discordance to calculate the difference between WGS and chip-based SNP genotyping^61^. Discordant SNVs, multi-allelic SNVs, non-variable SNVs, and those with <90% WGS call rates were removed leaving 146,076 SNP chip SNVs. The discordant SNVs were mostly comprised of SNVs within genomic regions with poor WGS mapping quality or those for which nearby variants alter the SNP chip genotype. Twenty-five additional genomes with average depths between 6.0x and 35.1x were genotyped at these 146,076 loci. Individual discordance was calculated after filtering the WGS genotypes by Genotype Quality (GQ = 0, 10 and 20). The percent discordance and percent WGS “no call” genotype according to GQ are presented in the Supplementary Fig. 2b.

### Breeds and variants analyses

In order to detect inacurate data and to validate the breed/species of each genome, we used a neighbor joining phylogeny comprised of variant positions and data (Supplementary Fig. 1). We compared 564 purebred, known mixed-breed, and unknown or uncertain heritage dogs having WGS data in the 722 WGS catalog to a dataset which was comprised of 1417 dogs from 193 breeds and nine wild canids (two golden jackal and seven wolves) that were previously published^4,11^, and 95 additionally genotyped samples available on Gene Expression Omnibus (GEO; https://www.ncbi.nlm.nih.gov/geo/; accession: GSE123368). After filtering variants for a GQ of 10 (vcftools filter -minQ 10)^61^, 145,470 SNVs were used to calculate distance matrix and run the phylogenetic analysis as described in Parker *et al.*^4^. Thirty dogs that did not group with the expected breed were marked “unknown”. In the end, the 722 genomes dataset was comprised of 538 dogs from 144 breeds with 54 breeds represented by three or more dogs. In order to annotate variants and run GWAS, we then kept only biallelic variants (SNV and indels) missing less than 10% of the individuals, for a total of 76.5 million variants using vcftools filters (–min-alleles 2 and–max-alleles 2–max-missing 0.9–minQ 20)^61^. Variants were then annotated using snpEFF version 4.3T^26^ and VEP 93^27^ with default parameters (Supplementary Table 1 and Supplementary Fig. 2c).

### GWAS

We included only samples with ≥10x coverage, selecting the two males and two females that had the deepest coverage when more than three individual by breed were available. All other samples were removed (including wild canids, village and feral dogs, unknown and mixed samples), leading to a dataset of 268 dogs representing 130 breeds. For each phenotype, we used average of the standard breed (male + female average). Standard breed weights (SBW), height (SBH) and life span were obtained from several sources: weights and height previously listed in Plassais et al.^17^, although they were updated if weights specified by the AKC^5^ were different. If the AKC did not specify SBW, SBH or life span, we used data from Atlas of Dog Breeds of the World^62^. SBW, SBH and life span were applied to all samples from the same breed. Phenotype information for fur length and furnishing were collected from Cadieu et al.^14^, bulky and muscled from Plassais et al.^17^ and these variables were encoded as NA/1/2 (NA = not applicable, 1 = not observed in the breed, 2 = observed in the breed). Behavior and tail shape values were collected from Vaysse et al. and Svartberg et al.^7,63^. We performed GWAS using GEMMA v0.94.1^28^ as linear-mixed model methods, removing variants with missing value > 1%, and correcting each analysis by sex and a relatedness matrix previously calculated. We used the multivariate linear mixed model^41^ available on GEMMA for life span analyses and included the SNP chip data for 746 genotyped dogs described in a previous paper^11^ (Supplementary Data 8). We first analyzed males and females separately, but observed no difference in male/female genotype distributions. Thus, further analyses utilized both sexes together. Of note, values shown on the X chromosome for *IRS4* and *IGSF1* at heterozygous genotypes correspond only to females (male are hemizygous on these loci). We used the Wald test to determine *P* values and Bonferroni correction was used to identify significant associations (cutoff = −log_10_ (0.05/number of variants) = 8.46). We removed the two outlier breeds (the Anatolian Shepherd Dog and the Tibetan Mastiff) and thus used 734 dogs to analyze the genotype distributions in the dog population for *LCORL*, *HMGA2*, *SMAD2*, *IGF1*, *IRS4* and *IGSF1*. *P* values were estimated by Mann–Whitney–Wilcoxon tests (*P < 0.05; **P < 0.01; ***P < 0.001). Manhattan, correlation and box-plots were constructed in R. For the 14 body size genes, the heridatibility of the most associated variant/mutation (h^2^) was calculated assuming Hardy-Weinberg proportions for the SNP genotypes as h^2^ = 2*p(1-p)*b^2^/σ^2^, where p was the allele frequency of the derived allele, b was the variant effect (regression coefficient estimated by GEMMA = beta), and σ^2^ was the phenotypic variance (=212.7 for SBW in this analysis).

### Sanger sequencing, qRT-PCR and protein alignment

Whole blood samples were collected into EDTA or ACD anticoagulant and genomic DNA was extracted using a standard phenol-chloroform extraction protocol. All procedures were reviewed and approved by the NHGRI Animal Care and Use Committee at the National Institutes of Health. Putative mutations (including those for *LCORL*, *ADAMTS9*, *HNF4G*, *R3HDM1*) were validated by Sanger sequencing. Targeted regions were amplified using polymerase chain reaction (PCR) with AmpliTaq Gold. PCR products were purified by ExoSap-It™ reaction (Affymetrix), and then sequenced using BigDye Terminator v3.1 (Applied Biosystems) on an ABI 3730 DNA analyzer. Sequence traces were analyzed using Phred/Phrap/Consed package^64–66^. RNA was extracted from testes using the RecoverAll™ Total Nucleic Acid Isolation Kit (Thermo Fisher Scientific) according to the manufacturer’s instructions. Reverse transcription was performed with 1 μg of total RNA using the High-Capacity cDNA Reverse Transcription kit (Applied Biosystems), according to the manufacturer’s instructions. *LCORL* cDNA was amplified and Sanger sequenced in ten dogs (small, medium and large breeds) using four primer pairs (Supplementary Data 7). To estimate the conservation of the LCORL proteins between dog and human we obtained both protein and gene sequences from Ensembl^67^ and used SIM^68^ and LALNVIEW^69^ to align sequences (Fasta sequences available in Supplementary Data 3). DNA-binding domains were predicted using InterPro^70^. To assess expression levels for all body size genes and candidate genes associated with ear phenotypes, we performed qPCR on diluted cDNA samples (1:20 dilutions from the 1–2 µg obtained after cDNA reverse transcription) using the Power SYBR Green PCR Master Mix kit (Applied Biosystems). qPCR reactions were run on the CFX384 Touch™ Real-Time PCR Detection System (Bio-rad) using standard procedures. For each experiment, we performed three biological replicates. Relative normalized expressions were determined using CFX Maestro™ Analysis Software (Bio-Rad). Primers for body size genes, ears phenotypes and *GAPDH* (reference gene) were designed using Primer3plus^71^ (Supplementary Data 7). For the *ESR1* gene we pooled results based on breed (two each of Cavalier King Charles Spaniels, English Springer Spaniels, German Shepherd dogs, Maltese, Yorkshire Terriers and three Golden Retrievers (Supplementary Data 7).

### Identification of positively selected genes

Evidence for selection was evaluated in five comparisons based on a subset of traits previously defined for GWAS: (1) long legs versus control; (2) bulky versus control; (3) small versus large; (4) drop ears versus control, and (5) large ears versus control. The SNPs from WGS catalog were extracted (–maf 0.05–min-alleles 2–max-alleles 2–remove-indels–keep) separately for each of the five analyses (Supplementary Data 9) using vcftools^60^. We retained the same set of samples used for GWAS (Supplementary Data 2). Beagle version 4.1^72^ was used to infer the haplotype phase. We then performed the XP-CLR (hgdp.uchicago.edu/Software/) test by using the following parameters: phased genotype input (p1), non-overlapping windows of 50 kb, a maximum of 600 SNPs allowed within each window (snpWin), and a correlation level cutoff of 0.95 to down-weight scores for highly correlated SNVs (corrLevel). The genetic map was assumed to be 1 cM/Mb. The distribution of XP-CLR scores showed robustness to the phase information (Supplementary Fig. 10).

The XP-EHH (http://hgdp.uchicago.edu/Software/) test^50^ was also performed, splitting the genome into non-overlapping segments of 50 kb using the maximum XP-EHH score of all SNPs within a window as the summary statistic. To take into account SNP density, we binned genomic windows according to their SNP numbers in increments of 200, combining all windows with SNVs ≥ 600 into one bin. Within each bin, for each window *i*, the fraction of windows with a value of the statistic greater than that in *i* is defined as the empirical *P* value, following the method previously reported^23^. The distribution of SNPs density in each window is provided in the Supplementary Fig. 11.

Case/control comparisons were reapeatedly performed using the randomly sampled 30 village dogs as a control data set to assess the robustness of the results (Supplementary Data 9). Village dogs exhibited significantly lower levels of LD across the genome compared to modern breeds^23^, which reflects frequent recombination events, making them a suitable outgroup for comparative analyses.

Regions in the top 1% of empirical distribution (XP-CLR) and with *P* values < 0.01 (XP-EHH) were designated as selective sweep regions, and candidate genes located within or in close proximity (distance < 100 kb) are considered positively selected genes. We excluded windows with <10 SNPs to prevent the addition of spurious signals. Given that the X chromosome has experienced different rates of evolution from autosomes, we defined the empirical top 1% of regions on the X separately. Finally, we used VCFtools v0.1.15^61^ to estimate the F_ST_ divergence statistic between populations.

### RNA-sequencing analysis

Data from 51 RNA-seq samples were obtained from the Sequence Read Archive (https://www.ncbi.nlm.nih.gov/sra) from previously published studies (Supplementary Data 6). FASTQ files were quantified to transcript per million (TPM) expression values using RSEM version 1.3^73^ (options: rsem-calculate-expression–num-threads 10–paired-end–bowtie2) with CanFam 3.1-Plus^59^ used as reference genome.

## Supplementary information

Supplementary Information

Description of Additional Supplementary Files

Supplementary Data 1

Supplementary Data 2

Supplementary Data 3

Supplementary Data 4

Supplementary Data 5

Supplementary Data 6

Supplementary Data 7

Supplementary Data 8

Supplementary Data 9

Supplementary Data 10

Supplementary Data 11

Source Data

## Data Availability

Genomes sequenced for this work, as well as all publicly available data used for alignment are available via the Short Read Archive (ncbi.nlm.nih.gov/sra; Bioproject number: PRJNA448733) and the complete data set (vcf file containing 91 million variants and 722 genomes) is available on NCBI. The source data underlying Figs. 2b, 3c, d and 4c and Supplementary Figs. 4 and 6 are provided as a Source Data file. All other data are contained within the article and its supplementary information.
