# Supplementary material for: Whole genome sequencing of canids reveals genomic regions under selection and variants influencing morphology
Source: Nat Commun. 2019 Apr 2;10:1489. doi: 10.1038/s41467-019-09373-w (PMC6445083; doi:10.1038/s41467-019-09373-w)
Supplement: Supplementary file 1 — Supplementary Information [file 41467_2019_9373_MOESM1_ESM.pdf]

# Whole Genome Sequencing of Canids Reveals Genomic Regions Under Selection and Variants Influencing Morphology

Plassais *et al*.

## SUPPLEMENTARY FIGURES

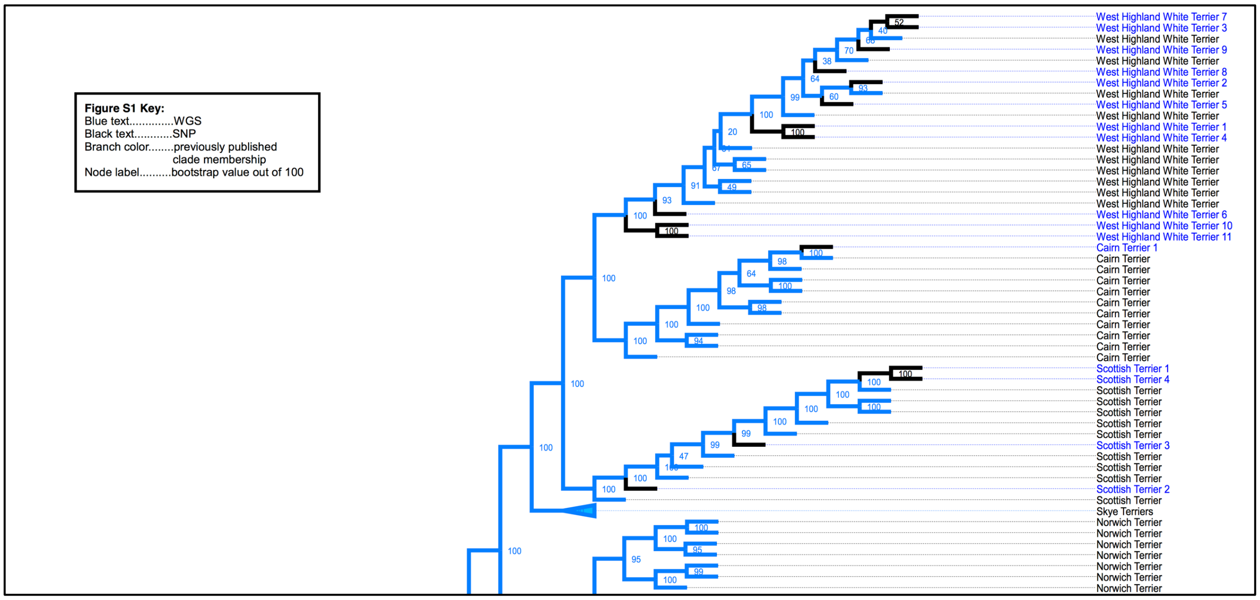


Example: Zoom in on the top of the phylogenic tree fully available on our website: https://research.nhgri.nih.gov/dog_genome/data_release/index.shtml

**Supplementary Figure 1. Neighbor joining tree of 2080 canids**. The tree is rooted with the Golden Jackal found at the bottom of the figure. Blue text highlights the WGS breed samples present in our canid catalog. The branches of the SNP genotyped individuals are colored to represent the clades identified in Parker et al[1]. Individuals from breeds without sequenced samples have been reduced to a single line. The number at each node is the bootstrap value out of 100 replicates.

a

| Category | Species / breeds | Number of genomes |
|---|---|---|
| Wild canids | Coyote, dhole, fox, jackal, wolves | 54 |
| Domestic dogs | 144 domestic breeds + 11 mixed samples (mixed dogs/breeds) | 538 |
| Village & Feral dogs | Village & indigenous dogs, dingo, New Guinea singing dogs | 104 |
| Unknown dogs | No breed information / not clustered with designated breeds* | 26 |
| * See Methods and S1 figure | Total | 722 |

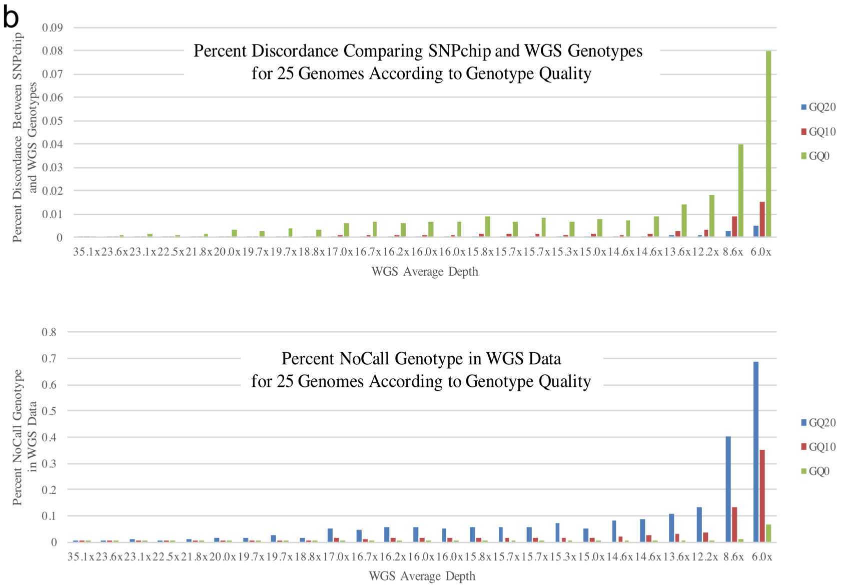


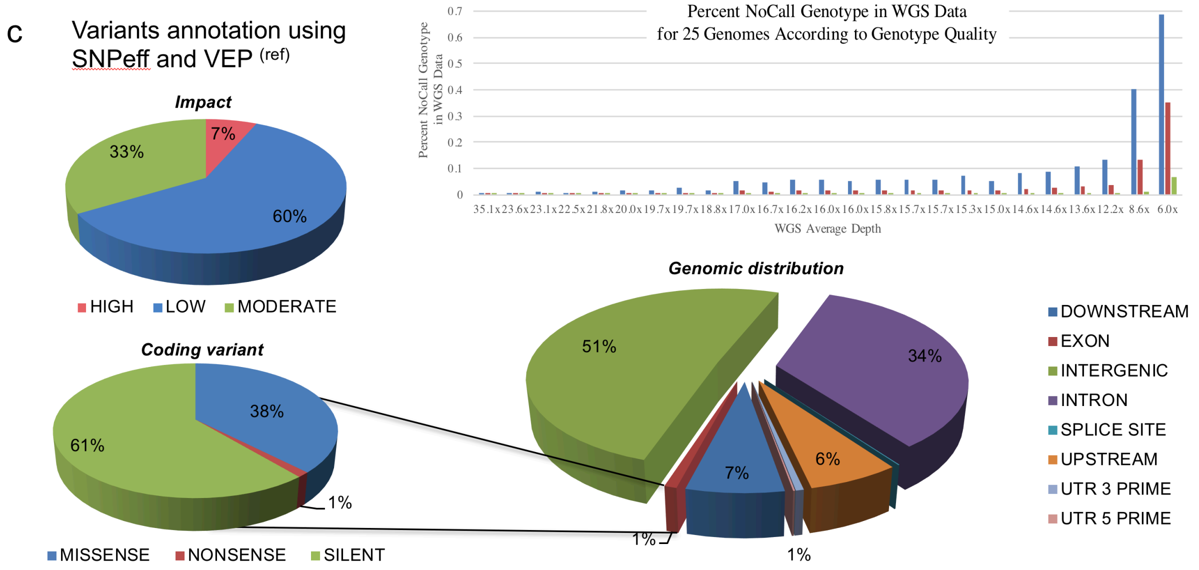


**Supplementary Figure 2. Summary of the canids catalog and the distribution of the 91 million variants.** (a) List of genomes used to build the catalog. (b) Percent discordance and WGS "no call" genotype according to genotype quality (GQ). Following these observations, we chose to filter WGS for a GQ of 20 keeping only WGS with an average depth >10x before performing GWAS. (c) Variants annotations combining two effect prediction tools: snpEFF version 4.3T[2] and VEP 93[3].

## Pipeline to run GWAS on the canids catalog using VCFtools[4] and GEMMA[5]

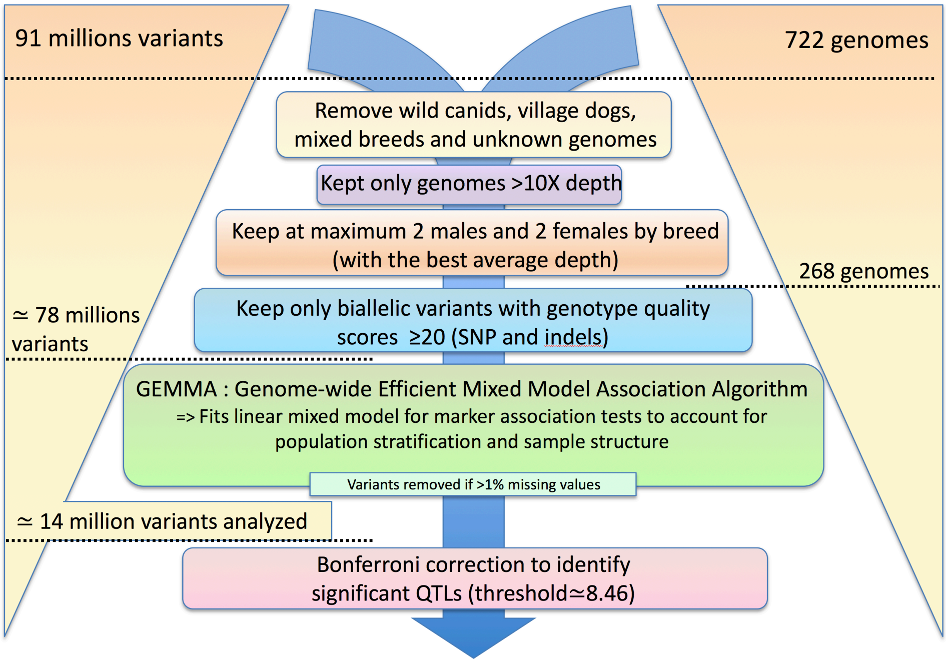


**Supplementary Figure 3**. **Whole genome sequencing assembly, variant calling and GWAS pipeline**. Sequence reads were aligned to the CanFam 3.1 reference genome (http://genome.ucsc.edu/cgi-bin/hgGateway?db=canFam3) using the BWA-MEM algorithm[6] (current version BWA 0.7.17) and sorted with SAMtools[7] (current version SAMtools 1.6). For non-PCR-free libraries, PCR duplicates were marked as secondary reads using PicardTools (http://github.com/broadinstitute/picard; current version PicardTools 2.9.2). GATK[8,9] (current version GATK 3.7) was used to perform local realignment around putative indels events using 714,278 variants previously published as a training set[10]. GATK base recalibration was performed using 172,254 Illumina Canine HD Chip positions and 2,738,537 dbSNP v131 variants. SNVs and small indels were called using GATK HaplotypeCaller, which first calls variants per-individual in gVCF mode with subsequent joint-calling utilizing all individuals[11]. Variant quality score recalibration was conducted with GATK best practices and default parameters for SNV and indels separately as follows. SNV recalibration: 172,254 Illumina Canine HD Chip variants (training, true, prior = 15); 2,738,537 dbSNP v131 variants (known, training, prior = 6); 3,627,539 published variants from Axelsson *et al*[10] (known, prior = 6). Indel recalibration: 714,278 variants as known, training and truth sets with a prior of six[10] and maxGaussians set to 4.

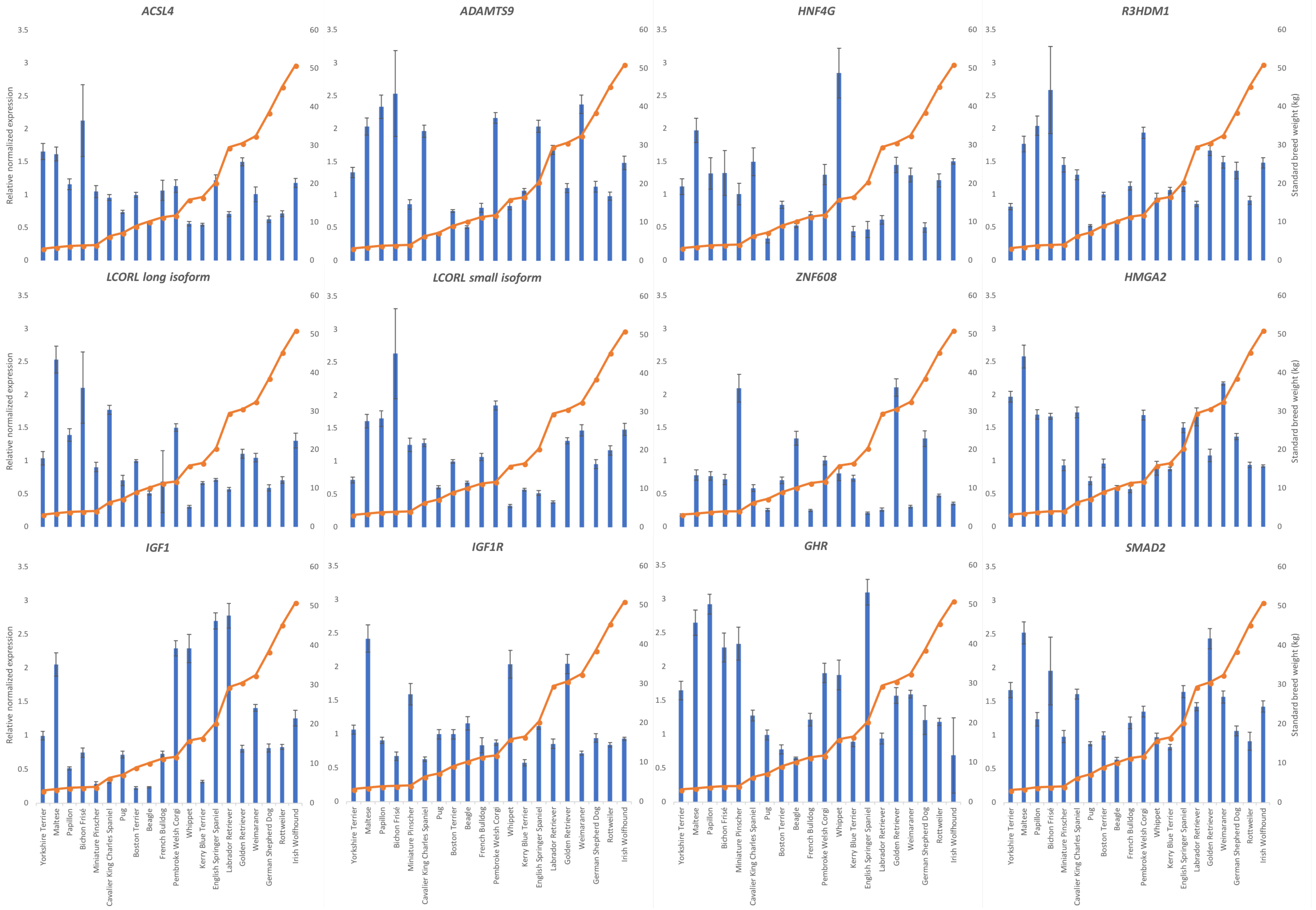


**Supplementary Figure 4. Relative normalized expressions of body size genes in testes.** No distinct correlations could be observed between relative normalized expressions level measured by qRT-PCR (blue) and standard breed weight (orange curve). Concerning *ACSL4*, *ADAMTS9*, *HNF4G*, *R3HDM1* and *ZNF608* associated with the bulky phenotype, our panel does not allow us to deduce any results. Indeed, our panel contains only one "bulky breed ("one Rottweiler). In addition, comparing these results to the RNA-Seq analyses described in the supplementary table 6, we can make the hypothesis that testes are probably not the best tissue to test the different *LCORL* isoforms and *GHR*, *HNF4G*, *IGF1* (weakly expressed), as well as *ADAMTS9-AS*, *IGSF1*, *IRS4* and *STC2* not detected by qRT-PCR. Source data are provided as a Source Data file.

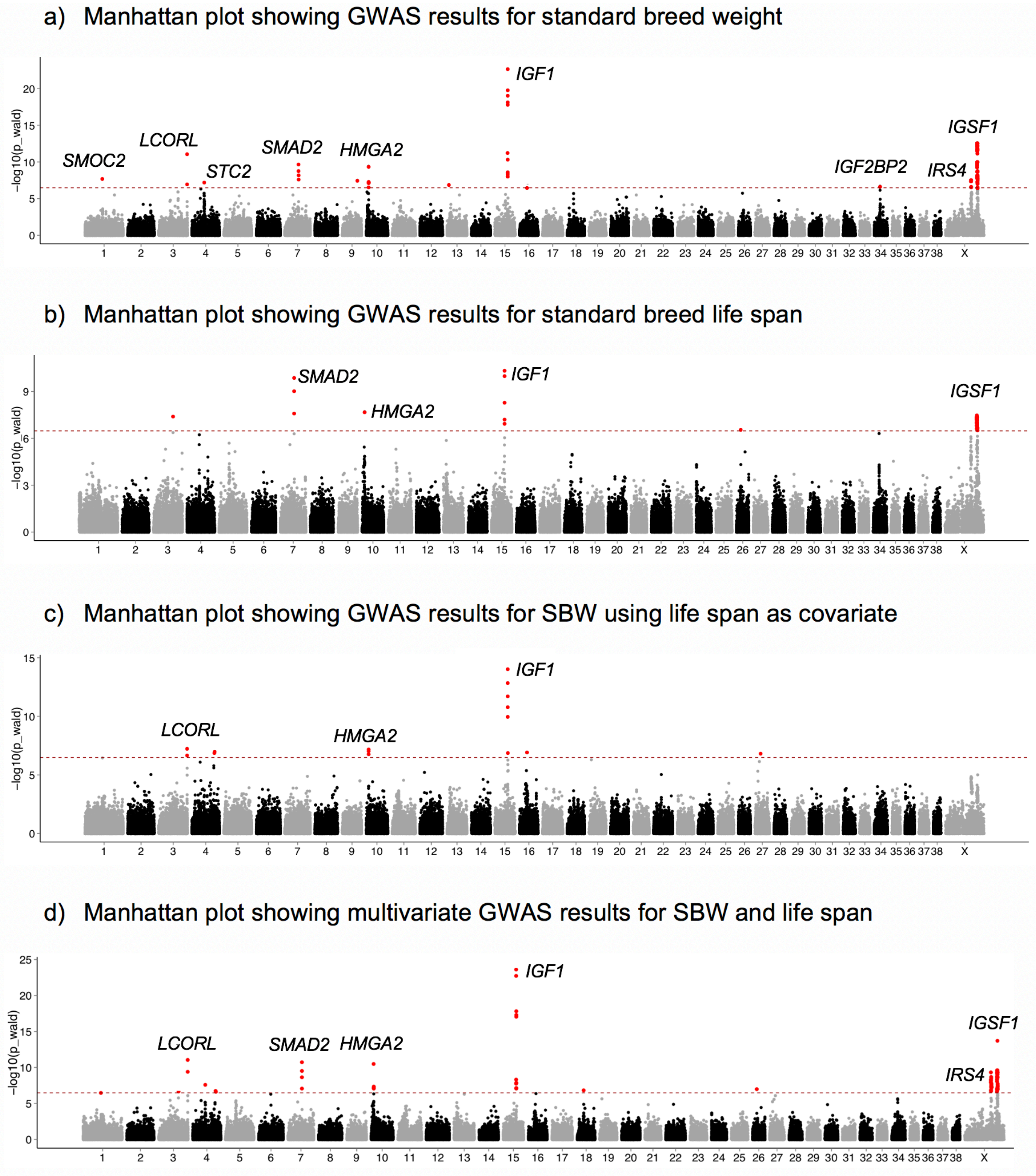


**Supplementary Figure 5. Manhattan plots showing GWAS for standard breed weight (SBW) and longevity using 714 dogs genotyped on Illumina canine HD SNP array (170k).** We observe that signals on CFA3, 7, 10, 15 and X loci are common between all GWAS, but decrease when life span is used as a covariable (c). As shown in figure D, multivariable GWAS confirmed that these loci are involved in both phenotypes (SBW and life span) tightly correlated, with higher p-values than in other GWAS (a, b and c). These results confirmed signals identified with the catalog of 722 canids genomes.

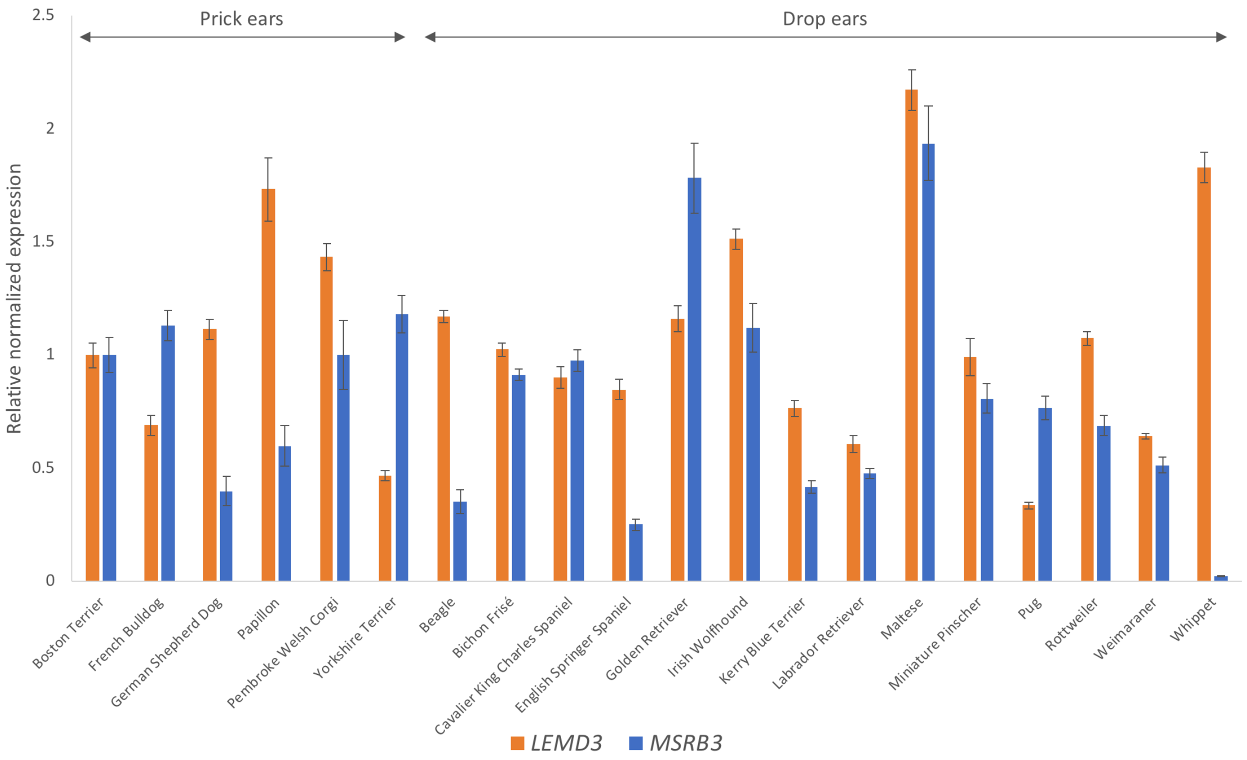


**Supplementary Figure 6. Relative normalized expressions of candidate genes associated with ears morphology.** No distinct correlations could be observed between ear morphology and the expressions level measured by qRT-PCR for *LEMD3* (orange) and *MSRB3* (blue). We did not detected signals for *WIF1 and* the mutated lncRNA on chromosome 10 identified in this paper, as well as the two candidate genes *RIMS1* and *KCNQ5* located on chromosome 12 associated with the "large and round ears" phenotype. Comparing these results to the RNA-Seq analyses described in the supplementary table 6, we can conclude, as expected, that testes are probably not the best tissue to analyze the ear morphology. Future investigations should focus on ear cartilages to check all gene expressions (*WIF1*, *LEMD3*, *MSRB3*, *RIMS1*, *KCNQ5*) and the lncRNA on chromosome 10. Source data are provided as a Source Data file.

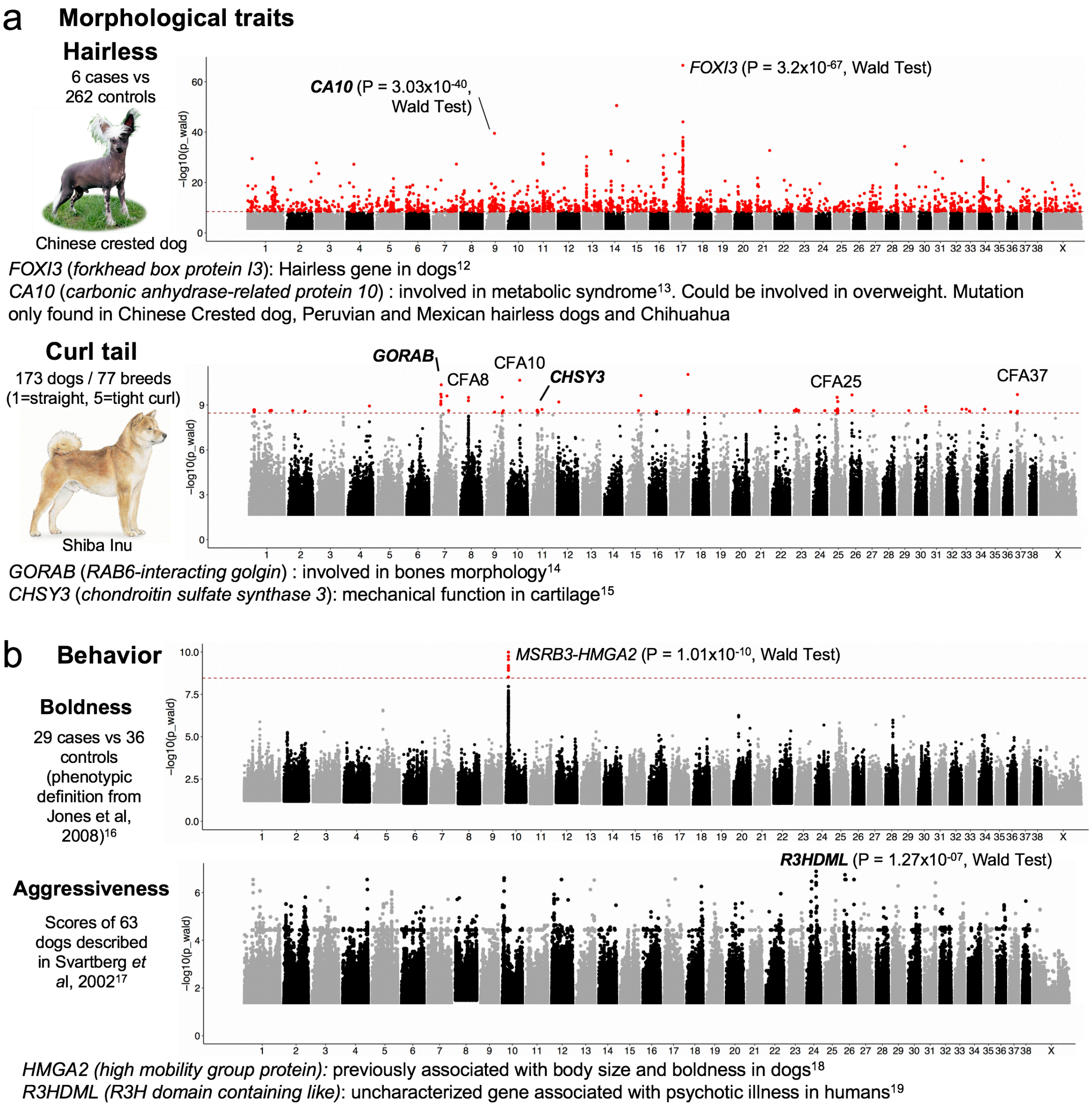


**Supplementary Figure 7. Manhattan plots showing association results for hairless, curl tail and behaviors.**

**Supplementary Figure 8. XP-CLR plots on candidate gene regions for body size trait, long legs (Sighthounds) and ears morphology.**

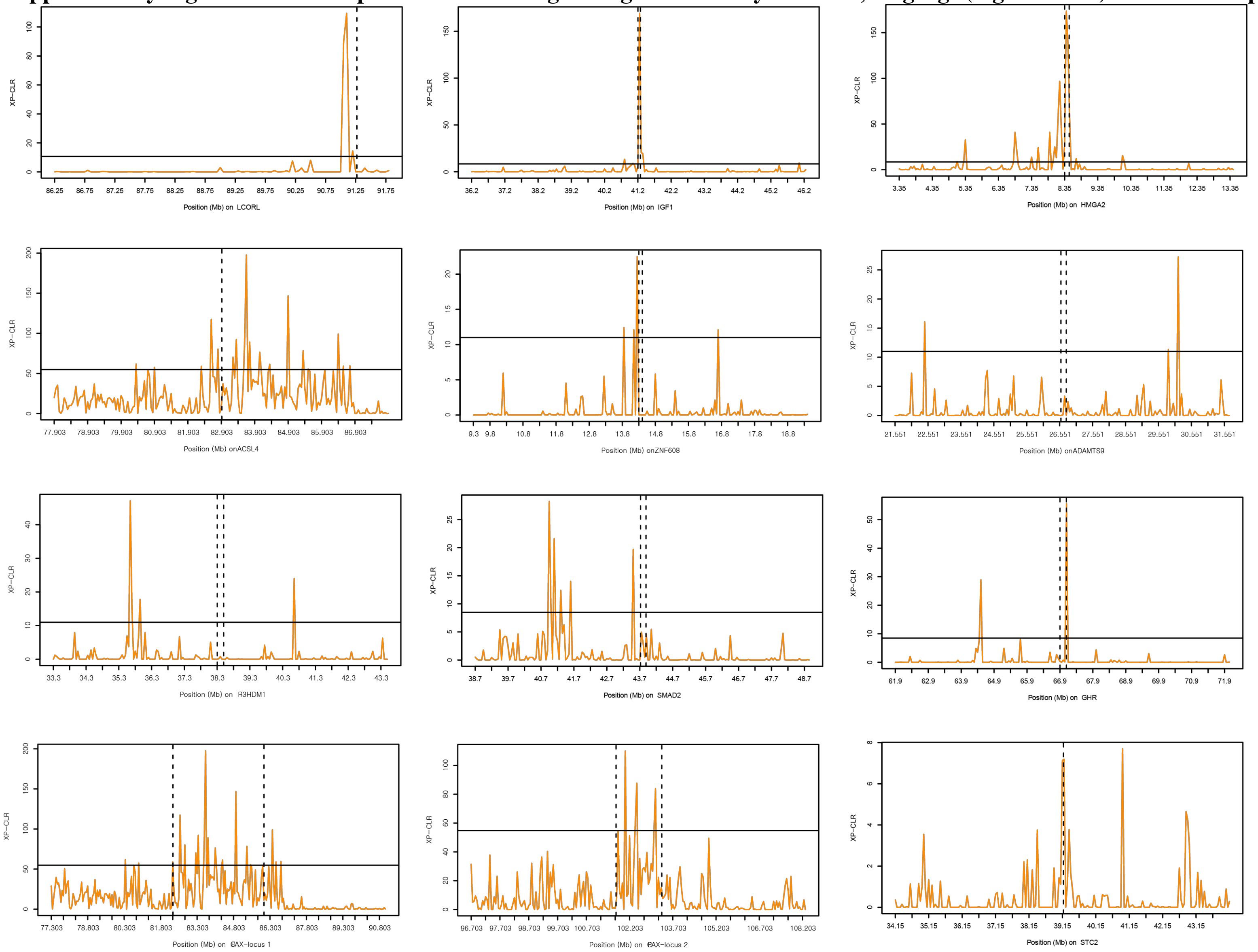

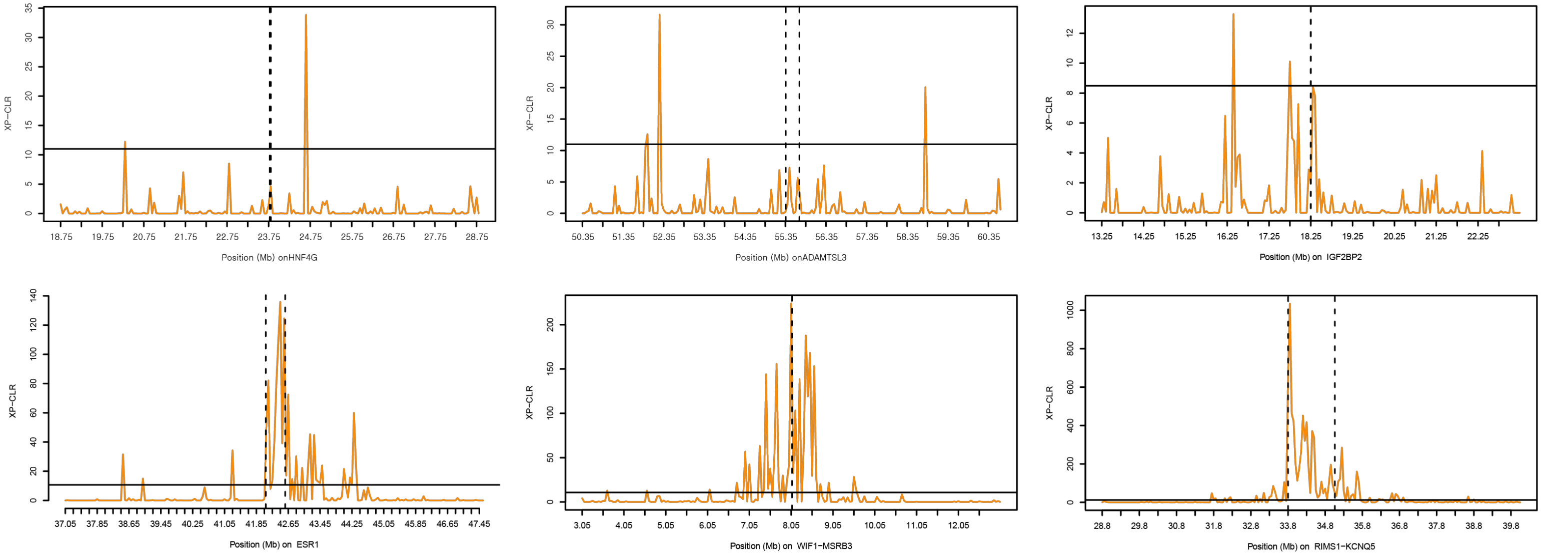


Vertical lines correspond to the candidate gene or variant identified by GWAS. Horizontal lines represent the empirical top 1% of genomic regions. Most of the examples illustrate the human-driven selection pressure to create breeds harboring a particular phenotype like the small size or the drop ears. The values and comparison with village dogs are also presented in the supplementary table 10.

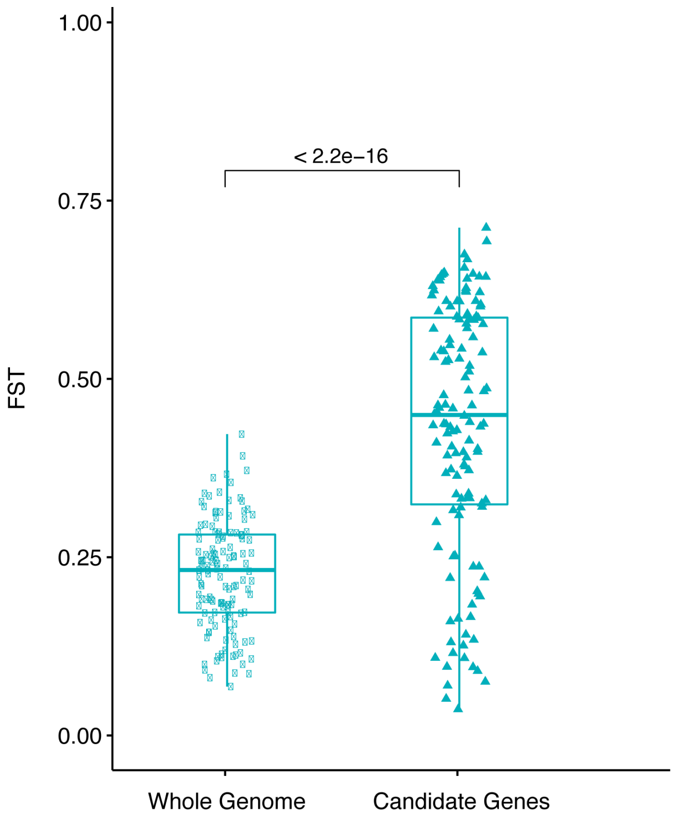


**Supplementary Figure 9. Boxplot of FST measured within candidate genes and whole genome between small and large breeds.** As expected, we observed a significant difference when we run selection scans on the entire genome or only focused on the 14 body size genes identified by GWAS and described in this paper.

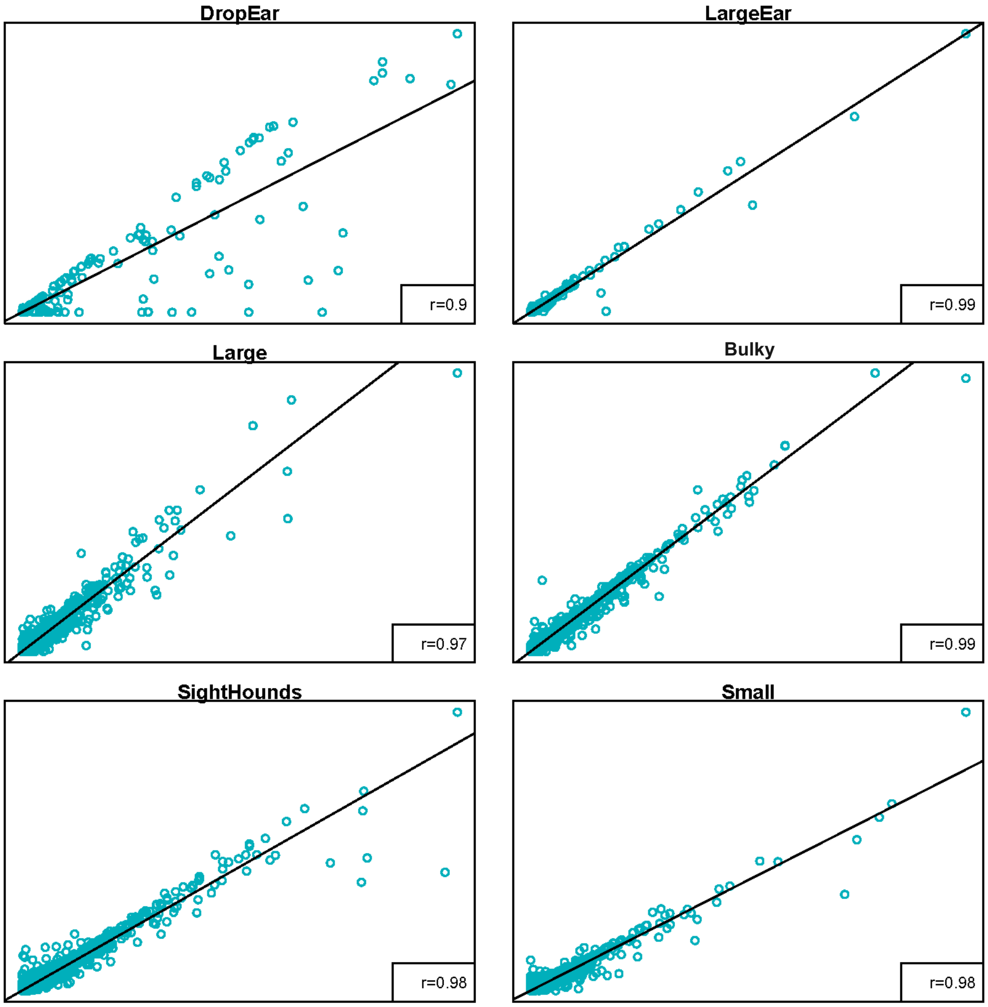


**Supplementary Figure 10. Distribution of XP-CLR scores based on phased *versus* unphased genotypes.**

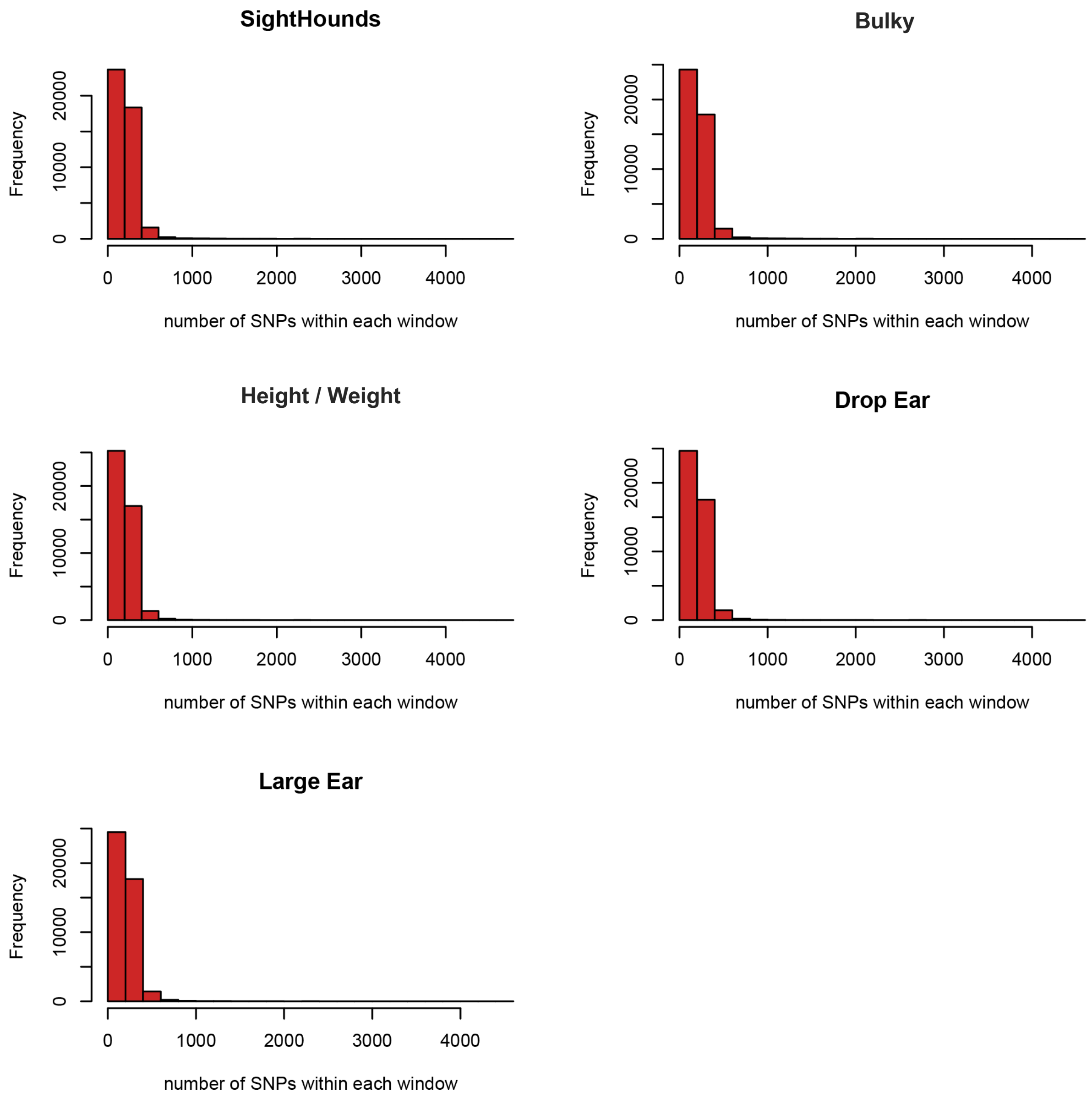


**Supplementary Figure 11. Distribution of number of SNPs within each 50kb window.**

# SUPPLEMENTARY TABLES

## Supplementary Table 1. Summary of variants detected in the 722 canids genomes using SnpEFF[2] and VEP[3]

| | SnpEFF | | VEP | |
|---|---|---|---|---|
| | Number | % | Number | % |
| HIGH | 69696 | 6.6% | 60527 | 6.6% |
| LOW | 629883 | 59.9% | 549222 | 52.2% |
| MODERATE | 352515 | 33.5% | 311387 | 29.6% |
| MISSENSE | 332559 | 37.6% | 297671 | 37.6% |
| NONSENSE | 11273 | 1.3% | 9403 | 1.2% |
| SILENT | 540063 | 61.1% | 484307 | 61.2% |
| DOWNSTREAM | 6318075 | 6.1% | 5637219 | 6.5% |
| EXON | 883895 | 0.8% | 791381 | 0.8% |
| INTERGENIC | 55256838 | 53.1% | 41872987 | 40.2% |
| INTRON | 34330953 | 33.0% | 31237519 | 30.0% |
| SPLICE_SITE_REGION | 97320 | 0.1% | 81286 | 0.1% |
| UPSTREAM | 6544634 | 6.3% | 5849265 | 5.6% |
| UTR_3_PRIME | 565967 | 0.5% | 511288 | 0.5% |
| UTR_5_PRIME | 139638 | 0.1% | 112603 | 0.1% |

**Supplementary Table 2. List of the breeds genotyped on the most associated SNP on *ESR1* locus using the Illumina HD canine SNP array (170k)**

A/A: Homozygote for the ancestral allele (blue); D/D: Homozygous for the derived allele (red);
A/D: Heterozygous (yellow)
- : Phenotype not applicable

| | | | chr1:42518863 | | |
|---|---|---|---|---|---|
| **Breed** | **Nb dogs** | **longlegs** | **A/A** | **A/D** | **D/D** |
| Afghan Hound | 10 | yes | | 1 | 9 |
| Akita | 10 | no | 1 | 2 | 7 |
| Alaskan Malamute | 10 | - | 5 | 5 | |
| American Cocker Spaniel | 10 | - | 10 | | |
| Anatolian Shepherd Dog | 6 | no | 6 | | |
| Australian Shepherd Dog | 10 | - | 6 | 4 | |
| Australian Terrier | 10 | - | 6 | 3 | 1 |
| Basenji | 10 | - | 6 | 2 | 2 |
| Basset Hound | 10 | - | 10 | | |
| Beagle | 11 | - | 11 | | |
| Belgian Shepherd | 10 | - | 2 | 3 | 5 |
| Belgian Tervuren | 10 | - | 6 | 4 | |
| Bernese Mountain Dog | 10 | no | 10 | | |
| Black Russian Terrier | 4 | yes | | 2 | 2 |
| Bloodhound | 10 | - | 10 | | |
| Border Collie | 10 | - | 7 | 3 | |
| Borzoi | 10 | yes | | | 10 |
| Boston Terrier | 10 | - | 10 | | |
| Boxer | 10 | - | 10 | | |
| Briard | 10 | - | 6 | 3 | 1 |
| Brittany | 10 | - | 3 | 5 | 2 |
| Bulldog | 10 | - | 10 | | |
| Bullmastiff | 10 | no | 8 | 2 | |
| Bullterrier | 9 | - | 10 | | |
| Cairn Terrier | 10 | - | 10 | | |
| Cardigan Welsh Corgi | 10 | - | 10 | | |
| Cavalier King Charles Spaniel | 10 | - | 10 | | |
| Chihuahua | 10 | - | 8 | 2 | |
| Chinese Shar-Pei | 10 | - | 8 | 2 | |
| Chow Chow | 10 | - | 4 | 4 | 2 |
| Collie | 10 | - | 8 | 2 | |
| Dachshund | 10 | - | 10 | | |
| Doberman Pinscher | 10 | - | 7 | 3 | |
| Dogue De Bordeaux | 6 | no | 4 | 2 | |

| | | | | | |
|---|---|---|---|---|---|
| English Cocker Spaniel | 10 | - | 9 | | |
| English Mastiff | 10 | no | 5 | 5 | |
| English Springer Spaniel | 10 | - | 5 | 4 | 1 |
| Flat-Coated Retriever | 10 | - | 10 | | |
| French Bulldog | 10 | - | 10 | | |
| German Shepherd Dog | 10 | - | 10 | | |
| German Shorthaired Pointer | 10 | - | 8 | 2 | |
| Giant Schnauzer | 10 | - | 7 | 3 | |
| Glen of Imaal Terrier | 9 | - | 6 | 3 | |
| Golden Retriever | 10 | - | 8 | 2 | |
| Great Dane | 10 | yes | | 3 | 7 |
| Great Pyrenees | 10 | yes | 1 | 5 | 4 |
| Greater Swiss Mountain Dog | 6 | no | 6 | | |
| Greyhound | 10 | yes | 2 | 2 | 6 |
| Havanese | 10 | - | 7 | 3 | |
| Ibizan Hound | 10 | - | | 2 | 8 |
| Irish Water Spaniel | 10 | - | 10 | | |
| Irish Wolfhound | 10 | yes | | 4 | 6 |
| Italian Greyhound | 10 | - | 10 | | |
| Kuvasz | 10 | no | 4 | 6 | |
| Labrador Retriever | 10 | - | 8 | 2 | |
| Leonberger | 10 | no | | 1 | 9 |
| Miniature Bull Terrier | 10 | - | 10 | | |
| Miniature Pinscher | 10 | - | 10 | | |
| Miniature Poodle | 10 | - | 8 | 2 | |
| Miniature Schnauzer | 10 | - | 10 | | |
| Neapolitan Mastiff | 6 | - | 1 | 3 | 2 |
| Newfoundland | 10 | no | 7 | 2 | 1 |
| Norwich Terrier | 10 | - | 4 | 5 | 1 |
| Old English Sheepdog | 10 | - | 9 | 1 | |
| Papillon | 10 | - | 7 | 3 | |
| Pekingese | 10 | - | 6 | 4 | |
| Pembroke Welsh Corgi | 10 | - | 9 | 1 | |
| Petit Basset Griffon Vendéen | 10 | - | 10 | | |
| Pomeranian | 10 | - | 9 | 1 | |
| Portuguese Water Dog | 10 | - | 10 | | |
| Pug | 10 | - | 8 | 2 | |
| Rottweiler | 11 | no | 11 | | |
| Saint Bernard | 10 | no | 5 | 5 | |
| Saluki | 11 | yes | 2 | 2 | 7 |

| | | | | | |
|---|---|---|---|---|---|
| Samoyed | 10 | - | 10 | | |
| Scottish Deerhound | 10 | no | 10 | | |
| Scottish Terrier | 10 | - | 10 | | |
| Shetland Sheepdog | 10 | - | 10 | | |
| Shih Tzu | 10 | - | 8 | 2 | |
| Siberian Husky | 10 | - | 8 | 2 | |
| Staffordshire Bull Terrier | 10 | - | 10 | | |
| Standard Poodle | 10 | - | 9 | 1 | |
| Standard Schnauzer | 10 | - | 10 | | |
| Tibetan Mastiff | 6 | no | 1 | 5 | |
| Toy Poodle | 10 | - | 8 | 1 | 1 |
| West Highland White Terrier | 10 | - | 7 | 3 | |
| Whippet | 10 | - | 6 | 4 | |
| Yorkshire Terrier | 10 | - | 9 | 1 | |
| **Total** | 855 | 200 | 610 | 151 | 94 |

| | **General population** | **Long legs** | **Normal legs** |
|---|---|---|---|
| Ancestral allele frequency | 0.80 | 0.19 | 0.74 |
| Derived allele frequency | 0.20 | 0.81 | 0.26 |

**Supplementary Table 3. Validation of the mutation on CFA10 and genotype of the most associated variant on CFA12 associated with the shape of ears**

For CFA12, we genotyped the most associated marker identified by GWAS using the 722 WGS catalog.
A/A: Homozygote for the ancestral allele (blue); D/D: Homozygous for the derived allele (red);
A/D: Heterozygous (yellow)
- : Phenotype not applicable

| | | | | chr10:8070103 | | | chr12:34439600 | | |
|---|---|---|---|---|---|---|---|---|---|
| Breed | Nb dogs | Drop ears | Large ears | A/A | A/D | D/D | A/A | A/D | D/D |
| Afghan Hound | 10 | - | no | 3 | 4 | 3 | 3 | 7 | |
| Akita | 10 | no | no | 10 | | | 8 | 2 | |
| Alaskan Malamute | 10 | no | no | 10 | | | 9 | 1 | |
| American Cocker Spaniel | 10 | yes | yes | | | 10 | | | 10 |
| Anatolian Shepherd Dog | 6 | yes | no | 4 | 1 | 1 | 6 | | |
| Australian Shepherd Dog | 10 | no | no | 8 | 2 | | 10 | | |
| Australian Terrier | 10 | no | no | 10 | | | 10 | | |
| Basenji | 10 | no | no | 10 | | | 10 | | |
| Basset Hound | 10 | yes | yes | | | 10 | 1 | 5 | 4 |
| Beagle | 11 | yes | yes | | 2 | 8 | | | 11 |
| Belgian Shepherd | 10 | no | no | 10 | | | 10 | | |
| Belgian Tervuren | 10 | no | no | 10 | | | 10 | | |
| Bernese Mountain Dog | 10 | yes | - | | 2 | 8 | 3 | 4 | 3 |
| Black Russian Terrier | 4 | no | no | 3 | 1 | | 3 | 1 | |
| Bloodhound | 10 | yes | - | | | 10 | 10 | | |
| Border Collie | 10 | no | no | 6 | 4 | | 8 | 2 | |
| Borzoi | 10 | - | no | 10 | | | 10 | | |
| Boston Terrier | 10 | no | no | 10 | | | 9 | 1 | |
| Boxer | 10 | yes | no | 10 | | | 10 | | |
| Briard | 10 | yes | no | | 8 | 2 | 10 | | |
| Brittany | 10 | yes | no | | | 10 | 10 | | |
| Bulldog | 10 | - | no | 6 | 4 | | 10 | | |
| Bullmastiff | 10 | yes | no | 10 | | | 10 | | |
| Bullterrier | 9 | no | no | 10 | | | 9 | | |
| Cairn Terrier | 10 | no | no | 6 | 3 | 1 | 10 | | |
| Cardigan Welsh Corgi | 10 | no | yes | 8 | 2 | | | | 10 |
| Cavalier King Charles Spaniel | 10 | yes | yes | | | 10 | | | 10 |
| Chihuahua | 10 | no | no | 4 | 4 | 2 | 5 | 5 | |
| Chinese Shar-Pei | 10 | - | no | 10 | | | 9 | 1 | |
| Chow Chow | 10 | no | no | 10 | | | 10 | | |

| | | | | | | | | | |
|---|---|---|---|---|---|---|---|---|---|
| Collie | 10 | no | no | 10 | | | 10 | | |
| Dachshund | 10 | yes | yes | | 3 | 7 | | 3 | 7 |
| Doberman Pinscher | 10 | - | no | 5 | 3 | 2 | 10 | | |
| Dogue De Bordeaux | 6 | yes | no | | 2 | 4 | 6 | | |
| English Cocker Spaniel | 10 | yes | yes | | | 10 | | | 10 |
| English Mastiff | 10 | yes | no | 4 | 3 | 3 | 10 | | |
| English Springer Spaniel | 10 | yes | yes | | 2 | 8 | 2 | 4 | 4 |
| Flat-Coated Retriever | 10 | yes | no | | | 10 | 10 | | |
| French Bulldog | 10 | no | yes | 5 | 4 | 1 | | 2 | 8 |
| German Shepherd Dog | 10 | no | no | 10 | | | 9 | 1 | |
| German Shorthaired Pointer | 10 | yes | - | | | 10 | 5 | 4 | 1 |
| Giant Schnauzer | 10 | yes | no | 4 | 3 | 3 | 9 | 1 | |
| Glen of Imaal Terrier | 9 | no | no | 10 | | | 9 | | |
| Golden Retriever | 10 | yes | no | | 4 | 6 | 10 | | |
| Great Dane | 10 | yes | no | 1 | 2 | 7 | 7 | 3 | |
| Great Pyrenees | 10 | yes | no | | 3 | 7 | 10 | | |
| Greater Swiss Mountain Dog | 6 | yes | no | | 2 | 4 | 6 | | |
| Greyhound | 10 | - | no | 10 | | | 10 | | |
| Havanese | 10 | yes | no | 2 | 6 | 2 | 5 | 4 | 1 |
| Ibizan Hound | 10 | no | no | 3 | 6 | 1 | 8 | 2 | |
| Irish Water Spaniel | 10 | yes | no | | | 10 | 9 | 1 | |
| Irish Wolfhound | 10 | - | no | 10 | | | 10 | | |
| Italian Greyhound | 10 | - | no | 8 | 2 | | 9 | 1 | |
| Kuvasz | 10 | yes | no | 4 | 6 | | 8 | 2 | |
| Labrador Retriever | 10 | yes | no | 2 | 4 | 4 | 10 | | |
| Leonberger | 10 | yes | no | | | 10 | 8 | 2 | |
| Miniature Bull Terrier | 10 | no | no | 10 | | | 10 | | |
| Miniature Pinscher | 10 | - | no | 10 | | | 8 | 2 | |
| Miniature Poodle | 10 | yes | yes | 9 | 1 | | 2 | 1 | 7 |
| Miniature Schnauzer | 10 | - | no | 10 | | | 10 | | |
| Neapolitan Mastiff | 6 | yes | no | | 2 | 4 | 6 | | |
| Newfoundland | 10 | yes | - | | 2 | 8 | 3 | 3 | 4 |
| Norwich Terrier | 10 | no | no | 10 | | | 10 | | |
| Old English Sheepdog | 10 | yes | no | | 1 | 9 | 8 | 2 | |
| Papillon | 10 | no | yes | 9 | 1 | | 1 | 3 | 6 |
| Pekingese | 10 | - | - | 1 | 4 | 5 | | 3 | 7 |
| Pembroke Welsh Corgi | 10 | no | yes | 10 | | | | | 10 |
| Petit Basset Griffon Vendéen | 10 | yes | yes | | | 10 | 3 | 4 | 3 |

| | | | | | | | | | |
|---|---|---|---|---|---|---|---|---|---|
| Pomeranian | 10 | no | no | 8 | 1 | 1 | 10 | | |
| Portuguese Water Dog | 10 | yes | no | 2 | | 8 | 8 | 2 | |
| Pug | 10 | - | no | | | 10 | 10 | | |
| Rottweiler | 11 | yes | no | 7 | 2 | 2 | 10 | 1 | |
| Saint Bernard | 10 | yes | - | 2 | 3 | 5 | 5 | 5 | |
| Saluki | 11 | - | no | | 1 | 9 | 8 | 3 | |
| Samoyed | 10 | no | no | 10 | | | 10 | | |
| Scottish Deerhound | 10 | - | no | 10 | | | 8 | 2 | |
| Scottish Terrier | 10 | no | - | 10 | | | 2 | 5 | 3 |
| Shetland Sheepdog | 10 | no | no | 2 | 5 | 3 | 10 | | |
| Shih Tzu | 10 | yes | no | 5 | 4 | 1 | 2 | 6 | 2 |
| Siberian Husky | 10 | no | no | 10 | | | 10 | | |
| Staffordshire Bull Terrier | 10 | no | no | 10 | | | 10 | | |
| Standard Poodle | 10 | yes | yes | 1 | 1 | 8 | 10 | | |
| Standard Schnauzer | 10 | - | no | 6 | 4 | | 10 | | |
| Tibetan Mastiff | 6 | - | no | | 2 | 4 | 5 | 1 | |
| Toy Poodle | 10 | yes | yes | 8 | 2 | | 1 | 3 | 6 |
| West Highland White Terrier | 10 | no | no | 10 | | | 10 | | |
| Whippet | 10 | - | no | 10 | | | 8 | 2 | |
| Yorkshire Terrier | 10 | no | no | 10 | | | 10 | | |
| **Total** | 855 | 688 | 785 | 456 | 128 | 271 | 621 | 107 | 127 |

| | **Chr10:8070103** | | | **Chr12:34439600** | | |
|---|---|---|---|---|---|---|
| | **General population** | **Drop ears breeds** | **Prick ears breeds** | **General population** | **Large ears breeds** | **Normal ears breeds** |
| Ancestral allele frequency | 0.61 | 0.29 | 0.92 | 0.79 | 0.22 | 0.95 |
| Derived allele frequency | 0.39 | 0.71 | 0.08 | 0.21 | 0.78 | 0.05 |

## SUPPLEMENTARY REFERENCES


1. Parker, H. G. *et al*. Genomic analyses reveal the influence of geographic origin, migration, and hybridization on modern dog breed development. *Cell Rep*. **19,** 697–708 (2017)

2. Cingolani, P. *et al*. A program for annotating and predicting the effects of single nucleotide polymorphisms, SnpEff: SNPs in the genome of Drosophila melanogaster strain w1118; iso-2; iso-3. *Fly (Austin)* **6,** 80–92 (2012).

3. McLaren, W. *et al*. The Ensembl Variant Effect Predictor. *Genome Biol*. **17,** 122 (2016).

4. Danecek, P. *et al*. The variant call format and VCFtools. Bioinformatics 27, 2156–2158 (2011).

5. Zhou, X. & Stephens, M. Genome-wide efficient mixed-model analysis for association studies. *Nat. Genet*. 44, 821–824 (2012).

6. Li, H. & Durbin, R. Fast and accurate short read alignment with Burrows-Wheeler transform. *Bioinformatics,* 25, 1754–1760 (2009).

7. Li, H. *et al*. The Sequence Alignment/Map format and SAMtools. *Bioinformatics,* 25, 2078–2079 (2009).

8. McKenna, A. *et al*. The Genome Analysis Toolkit: a MapReduce framework for analyzing next-generation DNA sequencing data. *Genome Res*. 20, 1297–1303 (2010).

9. DePristo, M. A. *et al*. A framework for variation discovery and genotyping using next-generation DNA sequencing data. *Nat. Genet*. 43, 491–498 (2011).

10. Axelsson, E. et al. The genomic signature of dog domestication reveals adaptation to a starch-rich diet. *Nature,* 495, 360–364 (2013).

11. Van der Auwera, G. A. et al. From FastQ data to high confidence variant calls: the Genome Analysis Toolkit best practices pipeline. *Curr Protoc Bioinformatics,* 43, 11.10.1–33 (2013).

12. Drögemüller, C. *et al*. A mutation in hairless dogs implicates FOXI3 in ectodermal development. *Science,* 321, 1462–1462 (2008).

13. Tekola-Ayele, F. et al. Genome-wide association study identifies African-ancestry specific variants for metabolic syndrome. *Mol. Genet. Metab*. 116, 305–313 (2015).

14. Yang *et a*l. Examining tissue composition, whole-bone morphology and mechanical behavior of GorabPrx1 mice tibiae: A mouse model of premature aging. *J Biochem,* 65, 145-153 (2017).

15. Filipek-Górniok, B. et al. Expression of chondroitin/dermatan sulfate glycosyltransferases during early zebrafish development. *Dev. Dyn*. 242, 964–975 (2013).

16 Jones P, Chase K, Martin A, Davern P, Ostrander EA, *et al*. (2008) Single-nucleotide-polymorphism-based association mapping of dog stereotypes. *Genetics,* 179: 1033–1044.

17. Svartberg K and Forkman B (2002). Personality traits in the domestic dog (*Canis familiaris*). *Applied Animal Behaviour Science*, 79: 133–155.

18. Vaysse, A. *et al*. Identification of genomic regions associated with phenotypic variation between dog breeds using selection mapping. *PLoS Genet*. 7, e1002316 (2011).

19. Bigdeli, T. B. *et al*. Comprehensive gene-based association study of a chromosome 20 linked region implicates novel risk loci for depressive symptoms in psychotic illness. *PLoS ONE,* 6, e21440 (2011).
