# Supplementary material for: Whole genome sequencing of canids reveals genomic regions under selection and variants influencing morphology
Source: Nat Commun. 2019 Apr 2;10:1489. doi: 10.1038/s41467-019-09373-w (PMC6445083; doi:10.1038/s41467-019-09373-w)
Supplement: Supplementary file 2 — Description of Additional Supplementary Files [file 41467_2019_9373_MOESM2_ESM.pdf]

Description of Supplementary Data

Supplementary Data 1. Catalog of the 722 canids genomes (Excel file)

Supplementary Data 2. Phenotypes and dogs used for the sixteen GWAS performed using the 722 genomes (Excel file)

Supplementary Data 3. Canine LCORL isoforms identified in this project (FASTA format)

Supplementary Data 4. Allele distribution of the variants associated with morphological traits identified with the WGS catalog (Excel file)

Supplementary Data 5. Validation of mutations by Sanger sequencing on a panel of 468 samples representing 100 modern dog breeds and 13 wild canids (Excel file)

Supplementary Data 6. List of the breeds genotyped on Illumina Canine HD SNP array (170k) (Excel file)

Supplementary Data 7. RNA expressions (TPM) using previously published RNA-SEQ data available on SRA (Excel file)

Supplementary Data 8. Samples and primers used for Sanger sequencing, cDNA detections and qRT-PCR experiments (Excel file)

Supplementary Data 9. List of breeds and village dogs used for selective sweep analyses (Excel file)

Supplementary Data 10. Summary of XP-CLR analyses between domestic dog breeds and village dogs (Excel file)

Supplementary Data 11. Summary of XP-EHH analyses between domestic dog breeds and village dogs (Excel file)

Source data. Raw data containing protein sequences used for the Fig. 2b, Mann-Whitney-Wilcoxon test results used for Figure 3c-d, and all relative normalized expression data used for Figure 4c and the supplementary figures 4 and 6.
